# Targeting the deubiquitinase STAMBP inhibits NALP7 inflammasome activity

**DOI:** 10.1038/ncomms15203

**Published:** 2017-05-11

**Authors:** Joseph S. Bednash, Nathaniel Weathington, James Londino, Mauricio Rojas, Dexter L. Gulick, Robert Fort, SeungHye Han, Alison C. McKelvey, Bill B. Chen, Rama K. Mallampalli

**Affiliations:** 1 Department of Medicine, Acute Lung Injury Center of Excellence, University of Pittsburgh, UPMC Montefiore, NW 628, Pittsburgh, Pennsylvania 15213, USA; 2 Departments of Cell Biology and Physiology and Bioengineering, University of Pittsburgh, Pittsburgh, Pennsylvania 15213, USA; 3 Medical Specialty Service Line, Veterans Affairs Pittsburgh Healthcare System, Pittsburgh, Pennsylvania 15240, USA

## Abstract

Inflammasomes regulate innate immune responses by facilitating maturation of inflammatory cytokines, interleukin (IL)-1β and IL-18. NACHT, LRR and PYD domains-containing protein 7 (NALP7) is one inflammasome constituent, but little is known about its cellular handling. Here we show a mechanism for NALP7 protein stabilization and activation of the inflammasome by Toll-like receptor (TLR) agonism with bacterial lipopolysaccharide (LPS) and the synthetic acylated lipopeptide Pam3CSK4. NALP7 is constitutively ubiquitinated and recruited to the endolysosome for degradation. With TLR ligation, the deubiquitinase enzyme, STAM-binding protein (STAMBP) impedes NALP7 trafficking to lysosomes to increase NALP7 abundance. STAMBP deubiquitinates NALP7 and STAMBP knockdown abrogates LPS or Pam3CSK4-induced increases in NALP7 protein. A small-molecule inhibitor of STAMBP deubiquitinase activity, BC-1471, decreases NALP7 protein levels and suppresses IL-1β release after TLR agonism. These findings describe a unique pathway of inflammasome regulation with the identification of STAMBP as a potential therapeutic target to reduce pro-inflammatory stress.

## Abstract (web summary)

How NALP7 inflammasome formation is regulated is unclear. Here the authors show that STAMBP prevents lysosomal degradation of NALP7 and present BC-1471 as a potential therapeutic STAMBP inhibitor, showing it can reduce TLR-induced IL-1β production.

The innate immune system utilizes recognition of pathogen-associated molecular patterns (PAMPs) or damage-associated molecular patterns (DAMPs) for host defense against microbial infection and tissue repair from injury. Extracellular PAMPs and DAMPs bind Toll-like receptors (TLRs) on the cell surface triggering downstream signal transduction and gene transcription through nuclear factor κB activation. Intracellular PAMPs and DAMPs are sensed by Nod-like receptors (NLRs), leading to assembly and activation of the inflammasome to generate the endogenous pyrogen interleukin (IL)-1β (refs 1, 2).

The inflammasome is a multimeric protein complex that assembles in response to NLR activation^3^. The largest family of NLRs is composed of pyrin domain-containing NLRs (NLRPs or NALPs)^4^. NALP7 inflammasomes are composed of multiple heterotrimeric subunits each consisting of the NLR sensor molecule NALP7, the adaptor protein ASC and the effector protein caspase-1 (refs 5, 6). Aggregation of these subunits enables cleavage of pro-caspase-1 to active capase-1, which then cleaves pro-IL-1β into mature IL-1β, a validated inflammatory therapeutic target^7^. Prior studies have shown that knockdown of NALP7 suppresses IL-1β release^5^, suggesting NALP7 as a putative target for anti-inflammatory therapy. Apart from its role as an inflammasome constituent, neither the cellular handling of NALP7 nor its druggability has been described.

Ubiquitin is a ∼8 kDa protein that functions as a universal protein trafficking modulator. Ub is covalently attached to proteins by the action of ubiquitin ligase enzymes (E3 ligases), and ubiquitinated substrate proteins are then bound for other cellular compartments or may be degraded by the proteasome or lysosome based on the extent and configuration of Ub adduction^8^. For example, K63 polyubiquitin chains can target proteins to the lysosome, whereas K48 linkages for polyubiquitination often mark proteins for proteasomal degradation. We have previously targeted E3 ligases critical for inflammatory signalling with therapeutic efficacy in preclinical models of inflammatory disease^9^,^10^,^11^,^12^. The de-ubiquitinase enzymes (DUBs) remove ubiquitin from proteins and thus oppose the action of the E3 ligases. Approximately 90 distinct DUBs, subdivided into five families, are encoded by the human genome^13^. The dynamic balance between E3 ligase and DUB activity on a substrate protein is therefore a key regulatory checkpoint of a protein's abundance, location and function^14^.

A pivotal trafficking pathway in cells is the endosome-lysosome pathway, which shuttles ubiquitin-tagged proteins to the lysosome for degradation. Mono- or poly-ubiquitination at the Ub lysine-63 residue (K63 linkage) can trigger cargo protein recruitment by the endosomal sorting complexes required for transport (ESCRT) machinery^15^. Among ESCRT members is the signal transducing adaptor molecule (STAM), which interacts with and recruits ubiquitinated cargo to the endosomal pathway^16^. STAM is closely associated with STAM-binding protein, STAMBP (also known as the associated molecule with the SH3 domain of STAM or AMSH), a metalloprotease and a member of the Jab1/MPN metalloenzyme (JAMM) family of DUBs^17^. STAMBP can de-ubiquitinate and rescue early and late endosome cargo proteins from progressing to lysosome compartmentalization^18^. Further, the DUB activity of STAMBP modulates the ubiquitination of ESCRT proteins STAM and hepatocyte growth factor-regulated tyrosine kinase substrate (HRS), but does not affect their degradation or stability^19^,^20^.

Here we demonstrate a unique physiological model for NALP7 inflammasome regulation via the endolysosomal cellular trafficking machinery. NALP7 is ubiquitinated at lysine residues 288 and 290, whereupon STAM constitutively recruits Ub-NALP7 for endosomal passage and lysosomal degradation. After cellular stimulation with infectious stimuli, NALP7 is rescued from endolysosome sorting to permit inflammasome-dependent IL-1β cleavage and release in a process dependent on the DUB activity of STAMBP. Further, we introduce the small molecule BC-1471, which interrupts STAMBP-Ub-NALP7 interaction and suppresses IL-1β release in several complementary human inflammatory systems, including THP-1 monocyte/macrophages, peripheral blood mononuclear cells and lung organ culture. These findings are, to our knowledge, the first demonstration of inflammasome component trafficking and modulation through the endolysosome system and the first rationally designed pharmacological inhibitor of inflammasome activity that targets this salvage pathway.

## Results

### TLR agonists promote NALP7 dependent IL-1β release

**Figure 1:**
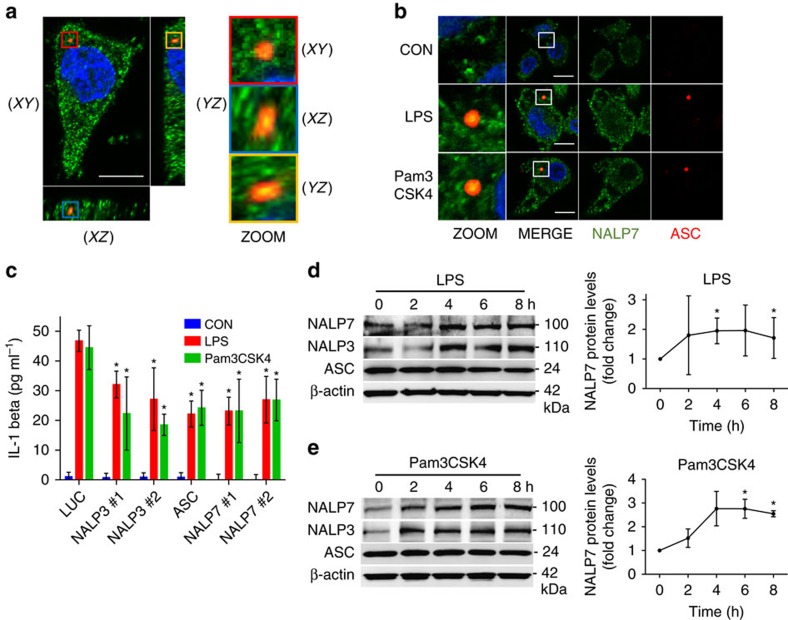
NALP7 forms an inflammasome to facilitate IL-1β release in response to LPS and Pam3CSK4. (**a**) A representative z-stack confocal microscopy image obtained by immunofluoresence co-staining of NALP7 (green), ASC (red) and DAPI (blue) in PMA-differentiated THP-1 cells (5 × 10^5^ per ml) following treatment for 4 h with LPS (100 ng ml^−1^). The 3D image is shown in XY, XZ and YZ coordinate planes. Scale bar, 10 μm. (**b**) Representative images obtained by immunofluorescence co-staining as above in PMA-differentiated THP-1 cells following treatment for 4 h with LPS (200 ng ml^−1^), Pam3CSK4 (100 ng ml^−1^) or vehicle control. Original magnification was × 100, Scale bar, 10 μm. (**c**) THP-1 cells transfected with *NALP3, ASC, or NALP7* siRNA followed by treatment with LPS (200 ng ml^−1^) or Pam3CSK4 (100 ng ml^−1^) for 6 h secreted less IL-1β as measured by ELISA compared to luciferase (LUC) siRNA control. Data shown as mean±s.d. (*n*=3). *P<0.05 compared to LUC control. (**d**,**e**) Immunoreactive NALP7 and NALP3 increased over time from lysates of THP-1 cells after (**d**) LPS (200 ng ml^−1^) or (**e**) Pam3CSK4 (100 ng ml^−1^) exposure for the indicated duration. *Right,* densitometric analysis of the NALP7 signal versus time, normalized to β-actin. Data shown as mean±s.d. (*n*=3). *P<0.05 compared to 0 h. (**c**) Two-way analysis of variance (ANOVA) with *post hoc* Dunnett's multiple comparisons test. (**d**,**e**) One-sample *t*-test (hypothetical value=1).

Prior *in vitro* studies demonstrate NALP7 inflammasome assembly in response to TLR1/2 agonists acylated lipopeptides (acLP), including the synthetic acLP, Pam3CSK4 (ref. 5). To investigate if the TLR4 ligand bacterial endotoxin also triggers NALP7 inflammasome assembly, human-derived THP-1 monocytic cells were differentiated to THP-macrophages with phorbol 12-myristate 13-acetate (PMA) and treated with lipopolysaccharide (LPS), Pam3CSK4 or vehicle control, and inflammasome formation and composition was evaluated by immunofluorescence microscopy. Inflammasome assembly in cells can be visualized by formation of a large ASC-speck^21^. Using z-stack confocal imaging, we demonstrate ASC-speck formation (red) co-localizing with NALP7 (green) in THP-macrophage cells treated with LPS or Pam3CSK4 but not under control conditions (Fig. 1a,b). To quantify NALP7 co-localization with ASC, we acquired z-stack images of cells with definitive ASC-specks and evaluated NALP7 co-staining by vector intensity profiling (Supplementary Fig. 1a). There was significant NALP7 co-staining within the ASC-speck in 90% (26/29) of LPS-treated cells containing an ASC-speck, while 82% (23/28) of cells exposed to Pam3CSK4 showed such co-localization. In the control condition, no cells were identified with an ASC-speck. This aggregation of NALP7 and ASC suggests that NALP7 forms an inflammasome in response to both LPS and Pam3CSK4.

NALP7 and ASC are each required for IL-1β maturation and release in response to acLP^5^. NALP3, perhaps the best characterized inflammasome-forming NLR and ASC are necessary for normal IL-1β release in cells treated with LPS^22^. To investigate whether NALP7 also contributes to LPS-mediated IL-1β release, THP-1 cells were treated with LPS and IL-1β release was evaluated in the setting of control siRNA or siRNA specifically targeting expression of NALP transcripts. In response to both LPS and Pam3CSK4, IL-1β release was significantly impaired when siRNA for NALP7 was present compared to control siRNA (Fig. 1c, Supplementary Fig. 1b). Cells treated with NALP3 or ASC siRNA effectively had reduced target proteins and secreted less IL-1β in response to either LPS or Pam3CSK4 (Fig. 1c, Supplementary Fig. 1b).

Since LPS increases the protein abundance of NALP3 in a concentration and time dependent fashion^23^, we hypothesized that TLR agonism may similarly increase NALP7 protein abundance. In THP1 cells, we observed a time- (Fig. 1d,e) and concentration- (Supplementary Fig. 1c,d) dependent increase in NALP7 protein in response to both LPS and Pam3CSK4 exposure.

### Bacterial endotoxin and Pam3CSK4 stabilize NALP7

**Figure 2:**
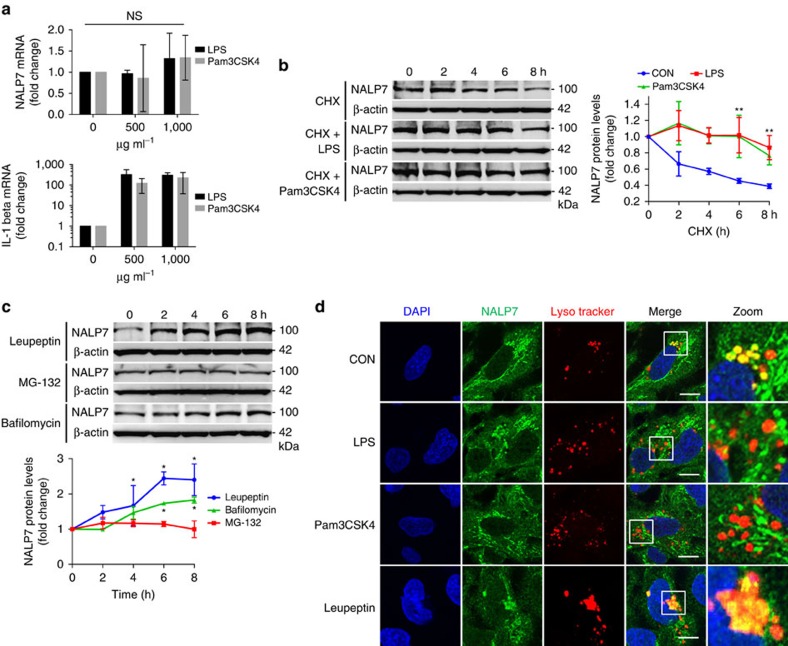
LPS and Pam3CSK4 alter NALP7 protein stability and trafficking. (**a**) NALP7 mRNA expression analysed by qPCR in THP-1 cells exposed to LPS or Pam3CSK4 for 16 h was unchanged compared to untreated control, whereas pro-IL-1β mRNA expression increased. Data shown as mean fold change±s.d. as determined by ΔΔCq analysis (*n*=3). (**b**) In cycloheximide (CHX) chase, NALP7 protein stability is increased in THP-1 cells exposed to LPS (200 ng ml^−1^) or Pam3CSK4 (100 ng ml^−1^) compared to vehicle control (CON) (**c**) NALP7 protein abundance increased over time when treated with leupeptin (50 μM) or bafilomycin (100 nM) but not MG132 (20 μM). (**b**,**c**) Densitometric analysis of the NALP7 signal versus time, normalized to β-actin. Data shown as mean±s.d. (*n*=3). *P<0.05 compared to 0 h or **P<0.01 versus CON. (**d**) Immunofluorescence co-staining of NALP7 (green), Lysotracker (red) and DAPI (blue) in Beas2B cells exposed to LPS (5 μg ml^−1^), Pam3CSK4 (2 μg ml^−1^), leupeptin (100 μM), or vehicle control for 2 h. Lysotracker (1:2,000) was added for the final 30 min of incubation before fixing and staining. Original magnification was × 100, Scale bar, 5 μm. Images are representative of three or more images captured for each condition (*n*=2). (**a**) One-sample *t*-test (hypothetical value=1). (**b**,**c**) Two-way analysis of variance (ANOVA) with *post hoc* Dunnett's multiple comparisons test.

To investigate the mechanism of LPS- and Pam3CSK4- mediated NALP7 induction, we first evaluated changes in transcription. TLR agonists did not significantly alter steady-state mRNA levels in THP-1 cells after 16 h exposure as determined by qPCR (Fig. 2a); however, these cells exhibit a significant increase in pro-IL-1β transcript. Since we did not observe increased NALP7 transcription, we next investigated NALP7 protein stability. LPS or Pam3CSK4 exposure for 16 h in the context of protein synthesis inhibition by cycloheximide (CHX) revealed significant NALP7 stabilization compared to control conditions, implying that NALP7 might be modulated post-translationally through its degradation (Fig. 2b).

Treatment with leupeptin (a lysosomal enzyme inhibitor) or bafilomycin (a vacuole acidification inhibitor) but not the proteasome inhibitor MG132 resulted in significant NALP7 accumulation (Fig. 2c), suggesting constitutive NALP7 degradation within the lysosome. To further investigate NALP7 trafficking and degradation by the lysosome, we used confocal imaging of human bronchial epithelial Beas2B cells, which express NALP7 and TLRs and permit easier lysosomal imaging compared to THP-1 cells given more abundant cytoplasm and expansive cell morphology. LPS, Pam3CSK4 or vehicle treated cells were labelled with the fluorescent lysosomal marker lysotracker and immunostained for NALP7. Unstimulated cells display robust NALP7 staining in the peri-nuclear region with co-localization of lysotracker labelling (Fig. 2d, upper panels, yellow). With LPS or Pam3CSK4 exposure, NALP7 staining was more peripheral and lysosomal co-localization was minimal. Leupeptin treatment also showed accumulation of NALP7 within the lysosome (lower panels). These data support the hypothesis that TLR signalling stabilizes NALP7 expression by decreasing its lysosomal trafficking and degradation.

### Ubiquitination acceptor sites modulate NALP7 stability

**Figure 3:**
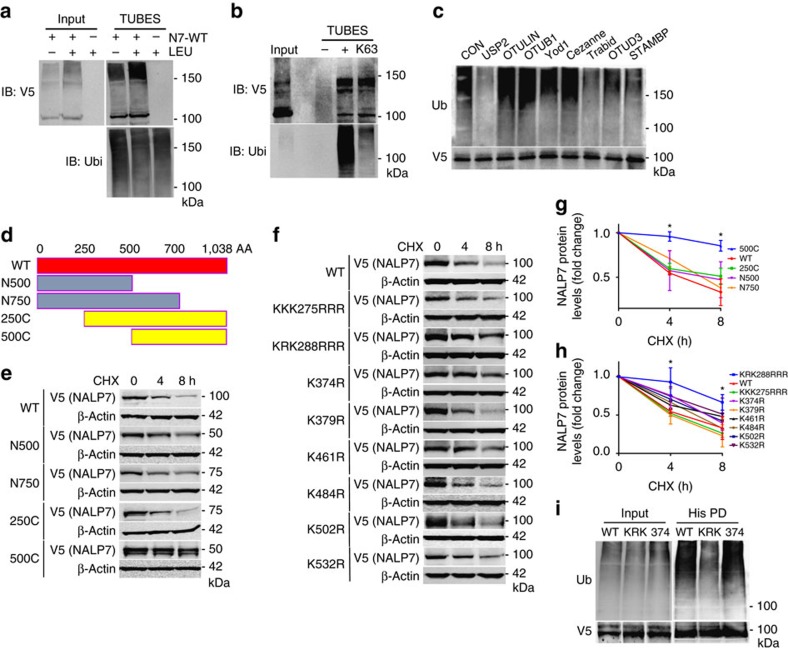
Identification of NALP7 ubiquitin acceptor sites. (**a**) TUBEs pulldown of ubiquitinated *NALP7*-V5 from denatured Beas2B cell lysates increased with leupeptin (LEU) (50 μM) treatment compared to untreated control. (**b**) Ubiquitinated *NALP7*-V5 was identified by immunoblotting after pulldown with non-selective TUBEs reagent and anti-K63 TUBEs from denatured Beas2B cell lysates. (**c**) UbiCREST assay. Immunopurified Ub-*NALP7*-V5 abundance decreased when incubated at 37 °C for 30 min with purified, recombinant USP2, Trabid, OTUD3 and STAMBP, but not OTULIN, OTUB1, Yod1 or Cezanne *in vitro*. (**d**) Mapping of NALP7 using truncation mutants. (**e**) Half life analysis of transfected wild-type and truncation mutant *NALP7*-V5 and (**f**) transfected K→R point mutant *NALP7*-V5 in Beas2B cells. (**g**,**h**) Densitometric analysis of (**g**) *NALP7* truncation mutant and (**h**) *NALP7* K→R point mutant signal versus time, normalized to β-actin. Data shown as mean±s.d. (*n*=2). *P<0.05 compared to wild-type using a two-way analysis of variance (ANOVA) with *post hoc* Dunnett's multiple comparisons test. (**i**) Immunopurified *NALP7*-V5 KRK288RRR (KRK) mutant showed decreased ubiquitination compared to wild-type or K374R mutant in Beas2B cells.

As substrate protein trafficking to the lysosome is often regulated by ubiquitination, we performed co-immunoprecipitation (co-IP) assays to confirm NALP7 ubiquitination. We used agarose-bound Tandem Ubiquitin Binding Entities (TUBEs) to pull down ubiquitinated proteins from Beas2B cells transiently transfected with a plasmid encoding V5-tagged NALP7. With this approach, we observed a slower migrating smear representing polyubiquitinated NALP7 (Fig. 3a). We hypothesized that NALP7 is conjugated with K63-linked ubiquitin given its lysosomal trafficking. When using TUBEs specific to K63-linked polyubiquitin, we again observed a slower migrating smear representing polyubiquitinated NALP7 (Fig. 3b). To further characterize NALP7 polyubiquitin chains, we performed a Ubiquitin Chain Restriction Enzyme Analysis (UbiCREST), which uses the linkage specificity of certain DUBs to characterize the polyubiquitin linkages conjugated to a substrate protein. When DUBs were added to immunopurified Ub-NALP7, we observed selective elimination of the high molecular weight Ub smear with USP2, Trabid and STAMBP (Fig. 3c). Because of its promiscuity in cleaving all linkage types, USP2 served as a positive control, confirming that the observed smear indeed represents polyubiquitinated substrates. On the basis of known linkage specificity of Trabid and STAMBP, NALP7 is conjugated with K63-linked polyubiquitin chains.

Protein ubiquitination occurs by formation of an isopeptide bond between a lysine acceptor site on a substrate protein and the incoming Ub moiety. The largest NALP7 isoform contains 69 lysine residues. To determine the NALP7 lysine acceptor site, we first cloned human NALP7 truncation mutants into a pcDNA3.1D/V5-His vector and evaluated protein stability of our ectopically expressed mutants following protein synthesis inhibition with CHX. We observed a significant increase in the protein stability of only the 500C NALP7 mutant (Fig. 3d,e,g), suggesting that a NALP7 ubiquitin acceptor site resides within 10 lysine residues between aa 250 and 500. Next, we generated point mutations, substituting arginine for lysine at these candidate acceptor sites. With CHX treatment, protein stability of the K288R and K290R (KRK288RRR) double mutant was significantly increased (Fig. 3f,h), similar to the effects of TLR agonists on wild-type NALP7 (Fig. 2b). Co-IP studies confirmed that the KRK288RRR NALP7 displayed decreased ubiquitination in comparison to wild-type or a NALP7 K374R mutant (Fig. 3i). These results suggest that KRK288/290 represent important lysine acceptor sites that modulate NALP7 cellular lifespan.

As ubiquitinated proteins bound for the lysosome often interact with the endosomal pathway and ESCRT machinery, we used siRNA to decrease expression of those ESCRT proteins with known ubiquitin interacting motifs. Knockdown of the ESCRT0 protein STAM, but not HRS, Tsg101 or EAP45, increased NALP7 protein abundance (Supplementary Fig. 2a); overexpression of *STAM* destabilized NALP7 protein (Supplementary Fig. 2b), suggesting that STAM is necessary for NALP7 degradation. As STAM is known to recruit ubiquitinated proteins to the endosomal pathway^16^, these findings strongly suggest that STAM plays a pivotal role in NALP7 cellular handling by recruiting Ub-NALP7 to the endosome for subsequent trafficking and lysosomal degradation.

### NALP7 stability is modulated through STAMBP DUB activity

**Figure 4:**
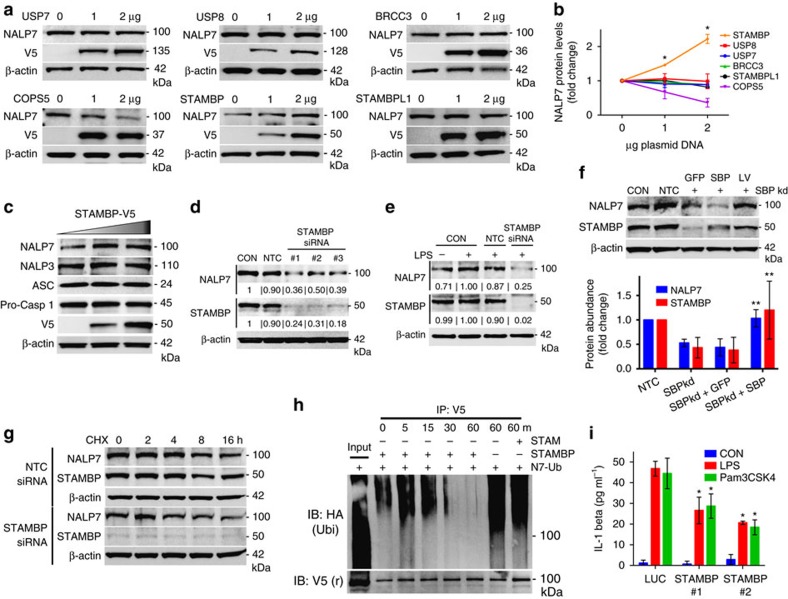
STAMBP modulates NALP7 protein stability and NALP7 inflammasome activity. (**a**) DUB screening in HeLa cells. Overexpression of plasmid-encoded *STAMBP,* but not other DUBs, increased endogenous NALP7 protein abundance. Note: V5 signal from BRCC3 is in the same location as actin causing actin signal distortion. (**b**) Densitometric analysis of NALP7 signal. Data shown as mean fold change±s.d. (*n*=2). *P<0.05 compared to empty vector control. (**c**) Overexpression of *STAMBP* plasmid increases NALP7 levels, but not other inflammasome constituents in Beas2B cells. (**d**) *STAMBP* knockdown with three separate siRNAs decreased endogenous NALP7 abundance compared to non-targeting control (NTC) siRNA in THP-1 cells. Relative abundance compared to control noted below each blot, as determined by densitometry using ImageJ software. (**e**) *STAMBP* knockdown with siRNA decreased endogenous NALP7 abundance compared to NTC siRNA after LPS exposure (500 ng ml^−1^ for 6 h) in THP-1 cells. Relative abundance noted as described previously. (**f**) *STAMBP* (SBP) expression by lentiviral transfection rescues NALP7 protein abundance in THP-1 cells treated with *STAMBP* siRNA. *Below,* densitometric analysis of relative NALP7 protein abundance as determined by ImageJ software. Data shown as mean±s.d. (*n*=4). **P<0.05 compared to STAMBP knockdown alone and STAMBP knockdown with GFP lentivirus rescue. (**g**) Endogenous NALP7 half-life is decreased with *STAMBP* knockdown by siRNA compared to NTC siRNA in Beas2B cells. (**h**) *In vitro* DUB assay with NALP7 substrate. Immunopurified *Ub-NALP7* substrate abundance decreased over time when incubated at 37 °C with purified recombinant STAMBP (200 nM) but not STAM (400 nM) or vehicle control. V5 is shown as a loading control. (**i**) IL-1β secretion following exposure to LPS (200 ng ml^−1^) or Pam3CSK4 (100 ng ml^−1^) for 6 h decreased in THP-1 cells transfected with *STAMBP* siRNA compared to control (luciferase, LUC) siRNA, as measured by ELISA. Data shown as mean±s.d. (*n*=3). *P< 0.05 compared to LUC control. (**b**) One-sample *t*-test (hypothetical value=1). (**i**) Two-way analysis of variance (ANOVA) with *post hoc* Dunnett's multiple comparisons test.

Since NALP7 seems to escape lysosomal trafficking and degradation when LPS or Pam3CSK4 are present, we hypothesized that deubiquitination of NALP7 would enhance its stability. Our prior data suggest that NALP7 is K63 polyubiquitinated, so we focused on DUBs with known K63 specificity. To identify the DUB associated with NALP7, we over-expressed plasmids expressing several candidate DUBs including BRCC3, which deubiquitinates NALP3 (ref. 24), COPS5, STAMBPL1, USP7 and the endosome-associated DUBs, STAMBP and USP8. In HeLa cells, immunoreactive levels of NALP7 were selectively increased after ectopic expression of a plasmid encoding the endosome-associated STAMBP (Fig. 4a,b). We recapitulated this finding in Beas2B cells and also observed that ectopic expression of STAMBP does not alter protein abundance of other inflammasome constituents NALP3, ASC or pro-caspase-1 (Fig. 4c). To test STAMBP effects on NALP7 stability in THP-1 cells, we silenced *STAMBP* expression with siRNA, which resulted in decreased NALP7 protein abundance (Fig. 4d). Further, LPS-induced increases in NALP7 levels were completely abrogated with *STAMBP*-targeting siRNA (Fig. 4e). Overexpression of STAMBP by lentiviral infection with a plasmid encoding STAMBP resulted in rescue of NALP7 levels (Fig. 4f). With CHX treatment, decay of NALP7 protein was accelerated by *STAMBP* knockdown (Fig. 4g). These findings suggest that STAMBP stabilizes NALP7 protein and is necessary for its upregulation in cells in response to LPS and Pam3CSK4.

Since STAMBP is known to function as a DUB, we developed a cell-free DUB assay to test STAMBP deubiquitination of NALP7 (ref. 25). When recombinant STAMBP was added to immunopurified Ub-NALP7, we observed a time-dependent reduction in the ubiquitin smear over 1 h when STAMBP was present (Fig. 4h). This finding supports that STAMBP is sufficient to directly deubiquitinate NALP7. While STAM modulates NALP7 and might possibly interact with the inflammasome component, it does not possess DUB activity, and we did not observe a similar reduction in the Ub-NALP7 substrate smear with inclusion of recombinant STAM in the assay mixture.

We next investigated whether the activity of STAMBP was necessary for normal cytokine release. IL-1β release was measured in THP-1 cells exposed to LPS and Pam3CSK4. We observed a statistically significant decrease in IL-1β secretion in cells transfected with siRNA targeting STAMBP compared to control siRNA (Fig. 4i; Supplementary Fig. 2c), suggesting that STAMBP activity is necessary for normal IL-1β maturation and release in response to the PAMPs tested.

### An antagonist of STAMBP DUB activity destabilizes NALP7

**Figure 5:**
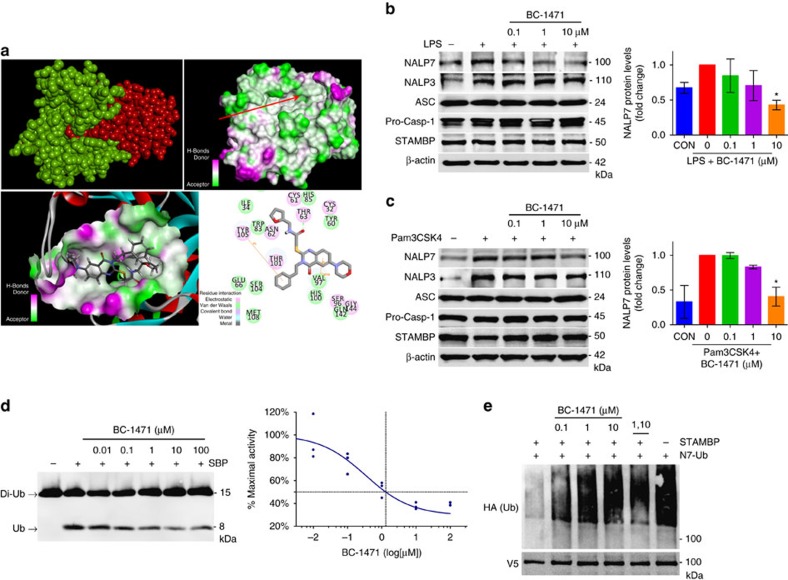
STAMBP inhibition with small molecule BC-1471 destabilizes NALP7 protein. (**a**) Top, left, a structural model of STAMBPL1 (green) interacting with Ub (red). Top, right, molecular charge mapping with red arrow pointing to the Ub-binding groove of STAMBP. Bottom, left, topological docking model of BC-1471 in the Ub-binding groove of STAMBP. Bottom, right, 2D mapping of key putative contact sites driving STAMBP and BC-1471 interaction. Amino acid numbers correspond to the residues of the published crystal structure of the catalytic domain of STAMBP (3RZU.pdb). (**b**,**c**) BC-1471 pre-treatment decreased NALP7 protein abundance after 6 h exposure to (**b**) LPS (200 ng ml^−1^) or (**c**) Pam3CSK4 (100 ng ml^−1^) compared to vehicle control (DMSO) in THP-1 cells. Right, densitometric analysis of the NALP7 signal for each condition relative to the treated positive control. Data shown as mean±s.d. (*n*=3). *P<0.05. (**d**) *In vitro* DUB assay with K63-linked di-Ub. BC-1471 inhibited cleavage of K63-linked di-Ub (200 nM) to mono-Ub by purified recombinant STAMBP (25 nM) incubated at 37 °C for 2 h in a concentration dependent manner. Graph depicts an inhibitor–response curve with IC_50_ determined by non-linear regression (*n*=3). (**e**) *In vitro* DUB with NALP7 substrate. Immunopurified *Ub-NALP7* substrate abundance was preserved with BC-1471 (10 μM) and 1,10-phenanthroline (5 mM) treatments, compared to vehicle control when incubated at 37 °C for 60 m with purified recombinant STAMBP (1 μM). V5 is shown as a loading control. (**b**,**c**) Two-way analysis of variance (ANOVA) with *post hoc* Dunnett's multiple comparisons test.

We designed and tested a small-molecule inhibitor of STAMBP in an attempt to destabilize NALP7 and down-regulate inflammasome activity. Using the STAMBP crystal structure^26^ (3RZU.pdb) and the structure of the nearly identical DUB domain of STAMBPL1 in complex with a di-Ub chain (2ZNV.pdb^27^), we used *in silico* molecular modelling to simulate the interaction between STAMBP and Ub (Supplementary Fig. 3a). Within STAMBP's DUB domain (Fig. 5a, shown in green), the Ub-binding groove interacts with the isopeptide-linked carboxy-terminal tail of the incoming Ub (shown in red). We hypothesized that molecular blockade of the Ub-binding groove might disrupt STAMBP's ability to deubiquitinate substrates. Targeting the STAMBP Ub-binding groove, we performed a high throughput *in silico* screen using a library containing greater than 500,000 experimental compounds. On the basis of our docking and best-fit analysis of suitable ligands, we selected 4-hydroxyquinazoline as a backbone and performed phenotypic screening of ten of its analogs in HeLa cells. Of those screened, BC-1471 was observed to exhibit the most potent ability to selectively decrease NALP7 abundance, but not NALP6 (Supplementary Fig. 3b). We next confirmed that BC-1471 also decreased endogenous NALP7 abundance in THP-1 cells in a concentration-dependent manner (Supplementary Fig. 3c). Hence, BC-1471 was selected as a tool compound for additional testing. In our docking model (Fig. 5a, lower panels), Thr63 forms electrostatic interactions with BC-1471, and Tyr 105 forms double π interactions with the benzene ring, with Val 97 forming σπ interactions with the quinazoline ring. Notably, orientation of this valine residue differs between STAMBP and STAMBPL1, which may impact interactions with BC-1471. In THP-1 cells, LPS- (Fig. 5b) and Pam3CSK4- (Fig. 5c) induced increases in NALP7 protein were suppressed by BC-1471 treatment; BC-1471 did not alter levels of immunoreactive NALP3, ASC or pro-caspase-1.

To validate the mechanism of action of our compound, we first performed a half-life experiment in Beas2B cells. BC-1471 significantly accelerated the degradation of NALP7 (Supplementary Fig. 3d) but increased NALP7 steady-state mRNA levels, perhaps as a compensatory effect (Supplementary Fig. 3e). To validate STAMBP as the target of our compound, we performed *in vitro* DUB assays. BC-1471 inhibited the activity of STAMBP to cleave recombinant di-Ub in a concentration-dependent manner with an half-maximal inhibitory concentration (IC_50_) of 0.33 μM (0.09–1.21 μM; Fig. 5d). In addition, we repeated the *in vitro* DUB assay with different concentrations of di-Ub and fit the results to enzyme inhibition models (Supplementary Fig. 3f). A comparison of fits determined that inhibition of STAMBP DUB activity by BC-1471 best fits a competitive inhibition model, whereby binding of BC-1471 inhibits binding of substrate to the active site of STAMBP. We next tested inhibition of STAMBP DUB activity specific to Ub-NALP7; here inclusion of BC-1471 blocked STAMBP mediated deubiquitination of Ub-NALP7 *in vitro* in a concentration-dependent manner (Fig. 5e), where BC-1471 performed comparably to the broad-spectrum metalloprotease DUB inhibitor 1,10-phenanthroline.

We performed a number of experiments to assess the specificity of BC-1471 to inhibit STAMBP but not other DUBs nor other metalloproteases. In a high throughput screen, BC-1471 did not significantly inhibit any of 38 other individual DUBs tested by inhibitor profiling (Supplementary Fig. 4a). Of note, this high throughput commercial screen utilized a ubiquitin-rhodamine substrate, which was not an optimal substrate for DUBs tested, especially those DUBs that demonstrate high K-63 linkage specificity, such as STAMBPL1. In a fluorescence-based activity assay, BC-1471 did not inhibit other metalloproteases MMP 1, 2, 8 or 9 (Supplementary Fig. 4b) underscoring its relative selectivity. Stability of the compound in our cell culture medium was confirmed by serial mass spectrometry demonstrating no significant degradation of the compound at 24 h (Supplementary Fig. 4c). Together, these data suggest that BC-1471 exerts DUB inhibitory activity on STAMBP in biochemical and cellular systems.

As STAMBP is known to modulate endosomal trafficking and degradation of ubiquitinated cargo^20^,^28^, we evaluated if STAMBP knockdown or inhibition could alter NALP7 trafficking through the endolysosome with microscopy. We quantified NALP7 co-staining using the late endosomal marker, mannose-6-phosphate receptor (M6PR), in the presence of leupeptin and observed statistically significant increases in NALP7 co-localization with M6PR by either STAMBP siRNA (Supplementary Fig. 5a,b) or BC-1471 (Supplementary Fig. 5a,c). These results are consistent with prior data demonstrating NALP7 degradation at the lysosome and STAMBP rescue of NALP7. Thus, by inhibiting NALP7 degradation (leupeptin) or by STAMBP small-molecule antagonism we can directly visualize NALP7 within the endolysosomal system.

### BC-1471 inhibits NALP7 inflammasome activity

**Figure 6:**
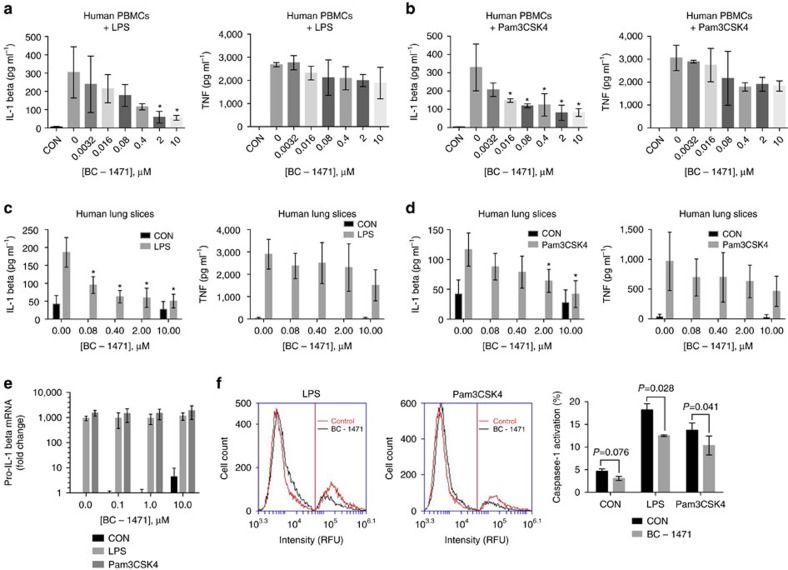
BC-1471 suppresses IL-1β secretion by inhibiting the NALP7 inflammasome. (**a**,**b**) IL-1β secretion following exposure to (**a**) LPS (10 ng ml^−1^) or (**b**) Pam3CSK4 (10 ng ml^−1^) for 2 h decreased in human PBMCs pre-treated with increasing concentrations of BC-1471 for 2 h, whereas changes in TNF secretion did not reach statistical significance. Data shown as mean±s.d. (*n*=3). *P<0.05 compared to LPS- or Pam3CSK4-exposed control. (**c**,**d**) IL-1β secretion decreased in human lung slices exposed to (**c**) LPS or (**d**) Pam3CSK4 pre-treated with BC-1471 as in **a**,**b**. Data shown as mean±s.d. (*n*=3). *P<0.05 compared to LPS- or Pam3CSK4-exposed control. (**e**) Increased pro-IL-1β mRNA expression in THP-1 cells after LPS (200 ng ml^−1^) or Pam3CSK4 (100 ng ml^−1^) exposure for 6 h is unchanged with BC-1471 pre-treatment. Data shown as mean fold change±s.d. as determined by ΔΔCq analysis (*n*=3). (**f**) FLICA assay. BC-1471 pre-treatment (10 μM) for 2 h decreased the population of THP-1 cells that stained positive for the carboxyfluorescein derivative substrate (that binds to activated caspase-1 in cells) following exposure to either LPS (200 ng ml^−1^) or Pam3CSK4 (100 ng ml^−1^) for 6 h. Cells were analysed for fluorscence by flow cytometry. Data shown as representative histograms, where the vertical lines show threshold fluorescence for cells with inactive (left) versus active (right) caspase-1, and graph that shows mean±s.d. (*n*=4). (**a**–**d**) One-way and (**e**,**f**) two-way analysis of variance (ANOVA) with post hoc Dunnett's multiple comparisons test.

We hypothesized that targeting of STAMBP DUB activity to destabilize NALP7 will decrease NALP7 inflammasome activity, namely secretion of mature IL-1β. To measure output from the NALP7 inflammasome in the presence of the small-molecule STAMBP inhibitor, we measured cytokine release from human peripheral blood mononuclear cells (PBMCs) by ELISA. PBMCs were pre-treated with increasing concentrations of BC-1471 prior to exposure to TLR agonists. IL-1β release was significantly suppressed by BC-1471 following exposure to LPS (Fig. 6a) or Pam3CSK4 (Fig. 6b). As a control, we measured TNF secretion from the same cells and observed a less profound decrease (Fig. 6a,b, right panels). We next tested BC-1471 in an ex vivo human model. Human lung slices obtained from explanted lungs deemed unsuitable for transplant were pretreated with BC-1471 as before. IL-1β secretion was again significantly decreased following exposure to LPS (Fig. 6c) or Pam3CSK4 (Fig. 6d) with less effect on TNF secretion (Fig. 6c,d, right panels), recapitulating our results from human PBMCs. Assessing toxicity of the compound, we treated THP-1 cells with BC-1471 and demonstrated a high half-maximal lethal concentration (LC_50_) of 106 μg ml^−1^ (Supplementary Fig. 4d), which was tenfold higher than the highest concentration used experimentally.

**Figure 7:**
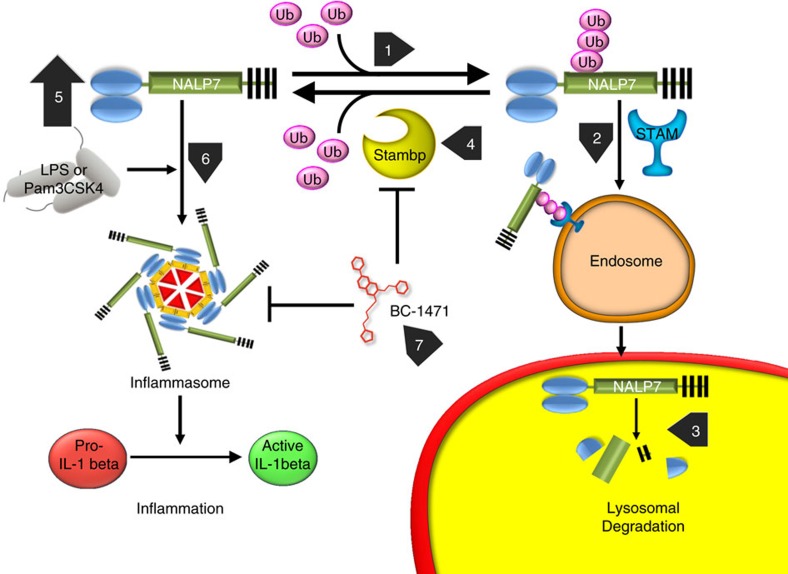
NALP7 inflammasome activity requires STAMBP DUB activity that is antagonized by BC-1471. In the absence of inflammatory stimuli, NALP7 is constitutively ubiquitinated (1) and bound by STAM as cargo for endosomal trafficking (2). Ub-NALP7 is shuttled to the lysosome for degradation (3). STAMBP deubiquitinates Ub-NALP7 facilitating NALP7 recue from degradation (4). With LPS or Pam3CSK4 exposure, NALP7 protein abundance increases (5) by decreasing endolysosomal trafficking in a process dependent on the DUB activity of STAMBP. The NALP7 inflammasome assembles in response to LPS or Pam3CSK4 (6) with subsequent IL-1β cleavage to its active form and inflammatory signalling. BC-1471 antagonizes STAMBP DUB activity (7), inhibiting NALP7 rescue, downstream of inflammasome assembly and IL-1β maturation.

To support the hypothesis that BC-1471 decreases inflammasome activity, we measured pro-IL-1β mRNA by qPCR in THP-1 cells pre-treated with the small-molecule inhibitor. Following exposure to LPS or Pam3CSK4, there was no significant difference in pro-IL-1β mRNA transcripts at any of the BC-1471 concentrations tested (Fig. 6e). We next tested caspase-1 activation in THP-1 cells using fluorochrome labelled inhibitors of caspases^29^ in the presence of TLR agonists and BC-1471. Activation of caspase-1 in response to either LPS or Pam3CSK4 was significantly suppressed by BC-1471 (Fig. 6f). Hence, small-molecule targeting of STAMBP destabilizes NALP7 protein and subsequently reduces activity of the NALP7 inflammasome to blunt IL-1β activation in response to TLR ligands (Fig. 7).

## Discussion

These studies demonstrate a unique ability of infectious-like stimuli, LPS and Pam3CSK4, to exploit the endosome-lysosomal trafficking machinery to initiate IL-1β driven inflammation. The new findings of this study are that (i) NALP7 is a ubiquitinated protein degraded within the lysosome, (ii) LPS and Pam3CSK4 preserve NALP7 protein by disrupting lysosomal trafficking, (iii) NALP7 abundance is modulated by the DUB, STAMBP and (iv) pharmacologic targeting of STAMBP deubiquitinase activity using a novel chemical entity abrogates induction of NALP7 levels in response to these PAMPs, thereby significantly suppressing IL-1β secretion. The efficacy of this rationally designed small-molecule inhibitor in targeting ubiquitin endolysosomal sorting underscores the importance of STAMBP as a central regulator of the NALP7 inflammasome that impacts the release of key cytokines implicated in human inflammatory disorders.

Our understanding of the biological role of NALP7 inflammasomes is evolving. Our data suggest that NALP7 is pro-inflammatory and that both acLP and endotoxin are stimuli for NALP7 inflammasome activation. NALP7 is known to sense acLP and is required for normal IL-1β maturation in response to this stimulus^5^. Prior data on LPS as a stimulus for NALP7 is mixed. Studies examining hydatidiform mole, a human disease associated with NLRP7 gene mutations, show that NALP7 is necessary for normal cytokine release in response to endotoxin^30^. Other in vitro studies have evaluated IL-1β release in inflammasome reconstitution systems that overexpress NALP7 in non monocytic cells^30^ or overexpress truncated NALP7 proteins and observed decreased cytokine output^31^. Our data suggest that functional NALP7 is necessary for normal IL-1β release in response to both LPS and Pam3CSK4 in human monocytic cells, supporting a pro-inflammatory role for NALP7, possibly making it a desirable anti-inflammatory therapeutic target.

The observations that NALP7 is ubiquitinated and trafficked through the endosome-lysosome pathway further highlight the importance of ubiquitin and the emerging role of endosomes in regulating cellular inflammation. Ubiquitination of inflammasome components tags substrate proteins for degradation or alternatively provides an activation signal^32^. In our model, K63 polyubiquitination of NALP7 results in endolysosomal trafficking and decreased protein abundance. Recent studies have suggested that NOD1 and NOD2, NLRs that activate NF-κB signalling, interact with their respective ligands at the membrane of the early endosome^33^,^34^. Our findings that NALP7 abundance is modulated by the ESCRT-0 complex protein STAM and the DUB STAMBP suggest that NALP7 may similarly function at the early endosome and that, furthermore, NALP7 is processed through the endosomal pathway to the lysosome for degradation. This endosomal trafficking of NALP7 is regulated by ubiquitin processing. Unlike NALP7, regulation of the NALP3 inflammasome by E3 ligases and DUBs has been demonstrated previously^23^,^24^,^35^, and the ubiquitination events and mediators prior to endosome recruitment are almost certainly an important means of NALP7 regulation. Further study is warranted to identify the E3 ligase(s) associated with NALP7 relevant for inflammation signalling.

Here we identify the DUB, STAMBP, as a critical regulator of NALP7 inflammasome activity. STAMBP is sufficient to deubiquitinate NALP7 and is necessary to stabilize NALP7 protein in cells exposed to LPS or Pam3CSK4. STAMBP is known to interact with ESCRT proteins to modulate the endolysosomal sorting and degradation of membrane receptors such as the epidermal growth factor receptor (EGFR) and the chemokine receptor CXCR4. Stimulation by EGF results in ubiquitination and trafficking of EGFR to the endosomal pathway through interactions with the ESCRT-0 complex^17^,^28^,^36^,^37^. Degradation of EGFR is enhanced by STAMBP knockdown following stimulation with EGF^19^. In contrast, basal expression of the CXCR4 receptor is increased by STAMBP knockdown or inhibition, in a process dependent on STAMPB interactions with the ESCRT-0 complex^20^. In the current study, STAMBP silencing decreases NALP7 levels constitutively, but has a more robust effect when cells are exposed to TLR agonists as a stimulus. Our finding that STAMBP knockdown reduces IL-1β secretion suggest that much of this effect is from NALP7 destabilization, but further study is needed to determine if endosomal trafficking and interaction with STAMBP is a required step for inflammasome assembly.

To our knowledge, BC-1471 represents a first-in-class rationally-designed anti-inflammasome drug. Using *in silico* design, we developed BC-1471 as a tool compound for use in our experimental models of inflammation. We mechanistically targeted a pharmacophore within the STAMBP ubiquitin-binding groove and not within the catalytic core, and based on efficacy and a lack of cytotoxicity in biologic testing selected BC-1471 from ten small molecules with a best predicted fit. Our intent was that this approach would give specificity for STAMBP antagonism and avoid off-target effects that could arise from inhibition of the highly conserved catalytic domain among JAMM/MPN+ family DUBs and other metalloproteases. Accordingly, BC-1471 failed to elicit inhibitory activity against any of the tested DUB family members or matrix metalloproteases, which share similar catalytic domains. Pending further testing, we cannot exclude off-target effects of the compound on closely related paralogues. Recent reviews of current DUB inhibitors have demonstrated a lack of specificity among published DUB inhibitors^38^ and stressed the importance of using appropriate substrates closely mimicking endogenous targets when developing a purported inhibitor^39^. As STAMBP activity is specific for K63-linked ubiquitin^38^, BC-1471 inhibition of STAMBP mediated cleavage of K63 linked di-Ub demonstrates physiologically relevant target validation for our compound. Further studies showing lack of off-target DUB inhibition by BC-1471 would justify its further use as a tool compound to investigate the role of STAMBP in inflammatory models. Additional pre-clinical testing of this STAMBP inhibitor, lead optimization, structure-activity relationships and investigative pharmacokinetic studies are needed.

Numerous recent studies have described various pharmacologic inhibitors of inflammasome activation^40^. However, the majority of these inhibitors are repurposed therapeutics, such as glyburide^41^,^42^, nucleoside reverse transcriptase inhibitors (NRTIs)^43^, parthenolide and Bay 11-7082 (ref. 44). Other compounds, such as β-hydroxybutyrate 45, type 1 interferon^46^ and MCC950 (ref. 47), have been shown to inhibit inflammasome activation, but their mechanism of action remains unclear. Our data suggest that BC-1471 reduces inflammasome activity by destabilizing NALP7 and thus suppressing IL-1β release. BC-1471 also appears to blunt pro-inflammatory caspase activation. This observation may be important because each activated caspase molecule facilitates the maturation of numerous IL-1β proteins^48^. Hence, effects of reduced caspase-1 activity are amplified with regard to cleavage of its substrate, IL-1β. In addition, it is important to note that NALP3 is not affected by BC-1471 and would remain available and active. The efficacy of BC-1471 validates NALP7, as well as STAMBP, as therapeutic targets in inflammatory signalling. Development of this inhibitor represents a previously undescribed therapeutic approach that might abrogate the injurious effects of unchecked IL-1β release in human inflammatory conditions.

## Methods

### Cell culture

THP-1, Beas2B and HeLa cell lines were purchased from the American Type Culture Collection (ATCC). Human PBMCs were from Sanguine Life Sciences. THP-1 cells and PBMCs were cultured in RPMI media supplemented with 10% FBS. THP-1 cells were differentiated into macrophages by treatment with 10 ng ml^−1^ of PMA (Sigma, P1585) for 16 h prior to further treatment. Beas2B cells were cultured in DMEM F-12 (DMEM/F12) supplemented with 5 or 10% FBS. HeLa cells were cultured in Eagle's minimum essential medium supplemented with 10% FBS. As indicated, THP-1 and Beas2B cells were treated with crude LPS (Sigma, L4391) or Pam3CSK4 (InvivoGen, tlrl-pms). For half-life studies, THP-1 cells were resuspended in 0% DMEM/F12 media prior to treatment with 10 μg ml^−1^ of cycloheximide (CHX) (Calbiochem, 239764) and Beas2B cells were treated with 40 μg ml^−1^ of CHX. THP-1 and Beas2B cells were treated with 20 μM of MG-132 (Sigma), 20 μM of bafilomycin (Sigma) or 100 μM of leupeptin (Sigma). Compounds (BC-1471) were solubilized at 5 mg ml^−1^ in DMSO before adding to the cells for up to 16 h.

### Confocal imaging

PMA-differentiated THP-1 macrophages or Beas2B cells were grown on glass-bottom culture dishes. Cells were treated as indicated and washed four times with PBS at the experimental endpoint. Cells were fixed using 2% paraformaldehyde, permeabilized with 0.05% saponin and then blocked with 10% goat serum in PBS with 0.05% saponin. Cells were probed with primary antibodies overnight, specifically: NALP7 (rabbit, Abcam, ab105405, 1:200 dilution), ASC (mouse, Santa Cruz, sc271054, 1:500 dilution) and M6PR (mouse, Abcam, ab2733, 1:400 dilution). Following washing, cells were incubated with Alexa Fluor conjugate secondary antibodies (Thermo Fisher, 1:1,000 dilutions) and briefly stained with DAPI. Cells were imaged using a Nikon A1 Confocal microscope at × 100 original magnification. Images were captured at 1024 × 1024 pixel density using Nikon Imaging Software Elements AR 3.2. Images were analysed for co-localization using CoLocalizer Pro Version 3.0.2 using published protocols^49^. We used ‘Auto Mode' for background correction and calculated all co-localization coefficients. For each biological replicate, we averaged coefficients from five images containing at least 20 cells for each experimental condition. For ASC Speck – NALP7 association studies, we used the Nikon software package for vector intensity quantification of single cells; we determined NALP7 signal intensity threshold to be 1500 RFUs (or higher) for significant co-staining, as average background NALP7 signal intensity was less than 1000 RFUs.

### siRNA transfection

All siRNA was purchased from the predesigned DsiRNA library from IDT, specifically: NALP3 (hs.Ri.NLRP3.13), ASC (hs.Ri.PYCARD.13), NALP7 (hs.Ri.NLRP7.13), STAM (hs.Ri.STAM.13), HRS (hs.Ri.HGS.13), TSG101 (hs.Ri.TSG101.13), VPS36 (hs.Ri.VPS36.13) and STAMBP (hs.Ri.STAMBP.13). Sequences for all DsiRNA are available in Supplementary Table 2. Firefly luciferase (FLuc-S1 DS) and a universal negative control (DS NC1) were purchased from IDT as negative controls. PMA-differentiated THP-1 cells (5 × 10^5^ cells per ml) were transfected with 40 nM siRNA duplexes using GenMute siRNA Transfection Reagent (SignaGen Laboratories) for 24 h. Cell were washed with fresh media and incubated for an additional 24–48 h before assay. Beas2B cells (70% confluent) were transfected with 20 nM siRNA duplexes using GenMute for 24 h. Cells were washed and fresh media was added before assay.

### Cytokine release assays

Following gene silencing or drug treatment, THP-1 cells or PBMCs were counted, normalized and divided into 96-well plates prior to treatment. Cells were stimulated with LPS or Pam3CSK4 for up to 6 h. Cytokine release was quantified from clarified culture supernatant using either human IL-1β (eBioscience, 88-7261) or human TNF (eBioscience, 88-7346) ELISA kits according to the manufacturer's protocol. Each experiment was replicated and performed in triplicate.

### Immunoblotting

Cells were lysed in RIPA buffer, sonicated and clarified by centrifugation. Lysates were diluted in SDS protein sample buffer. Proteins were separated by electrophoresis and transferred to a nitrocellulose membrane. Blots were blocked in 5% milk, followed by probing overnight with antibodies: NALP7 (rabbit, Abcam, ab105405), NALP3 (mouse, Adipogen, AG-20B-0014), ASC (rabbit, Adipogen, AG-25B-0006), HA (rabbit, Santa Cruz, sc-805), V5 (mouse, Invitrogen, R960), Ub (mouse, Life Sensors, VU-1 with glutaraldehyde pre-treatment per manufacturer's protocol), STAM (mouse, Santa Cruz, sc-133093), HRS (mouse, Santa Cruz, sc-271925), Tsg101 (mouse, Santa Cruz, sc-7964), EAP45 (mouse, Santa Cruz, sc-79931), caspase-1 (mouse, R&D Systems, MAB6215), STAMBP (mouse, Santa Cruz, sc-271641) and NALP6 (rabbit, Abcam, ab116007). All antibody solutions were diluted 1:1,000, except NALP7 (1:500), HA (1:2,000), V5 (1:5,000) and caspase-1 (1:2,000). Following addition of secondary antibodies (1:2,000 dilution), membranes were developed using SuperSignal West Femto Chemiluminescent Substrate (Pierce) and imaged using Kodak imaging software. Single band intensity was quantified using ImageJ software. Original, uncropped blots can be found in Supplementary Fig. 6.

### Quantitative PCR

Total cellular mRNA was collected from cells using the RNeasy MiniKit (Qiagen), following the manufacturer's protocol. The mRNA was then used to create cDNA using the High-Capacity cDNA Reverse Transcription Kit (Applied Biosystems) according to the manufacturer's protocol. qPCR was performed using SYBR Select Master Mix (Applied Biosystems) according to the manufacturer's protocol with 20 ng cDNA as a template and primer concentration of 200 nM. Each biological replicate was performed in technical triplicate; data was analysed using the ΔΔCq method. qPCR primer sequences are available in Supplementary Table 1.

### Cloning and mutagenesis

The cDNA encoding HA-ubiquitin was a gift from Dr Yutong Zhao. NALP7, STAMBP, STAMBPL1, USP7, COPS5 and USP8 cDNA with complete protein coding gene sequences, all in pLX304 vector, were purchased from DNASU. Flag-HA-BRCC3 (Addgene plasmid #22540) was a gift from Wade Harper^50^. pFBD-ESCRT 0 (encoding STAM and HRS) was a gift from James Hurley (Addgene plasmid # 21499)^51^. The coding sequences from NALP7, STAM, STAMBP and BRCC3 plasmids as well as the NALP7 truncation mutants were cloned using the Phusion High-Fidelity DNA Polymerase kit (New England BioLabs). Site-directed mutagenesis was performed using the QuikChange II XL Site-Directed Mutagenesis kit (Agilent). All primers were purchased from Integrated DNA Technologies (IDT), and sequences are available in Supplementary Table 1. After cloning, cDNA was ligated into pcDNA3.1D/V5-His vector (Invitrogen) and constructs were verified by Sanger sequencing. Plasmids were overexpressed in HeLa cells using Turbofect transfection reagent (Thermo Scientific) or Beas2B cells using XtremeGene HP DNA Transfection Reagent (Roche) for 24–48 h before assay.

### TUBEs pulldown and immunoprecipitation

All ubiquitin immunoprecipitation was performed under denaturing conditions. Lysates were harvested in RIPA buffer and immediately heated to 95 °C for 5 min, followed by sonication and centrifugation. Ubiquitinated substrates were precipitated from lysates using agarose-bound Tandem Ubiquitin Binding Entities (TUBEs, Life Sensors, UM401) following the manufacturer's protocol. We used the non-specific TUBE1 (Life Sensors, UM401), based on the ubiquitin binding domain of the protein ubiquilin, and the K63 specific TUBE (Life Sensors, UM304), based on an engineered K63-linked peptide^52^. V5 immunoprecipitation was performed according to the protocol provided by the manufacturer with the modification that Protein A/G beads (Pierce) were used to bind the antibody. For STAM IPs, lysates were harvested in Buffer A (1 × PBS, 0.5% Triton X100, protease and phosphatase inhibitors), sonicated and centrifuged before rocking for 2 h with STAM antibody and 1 h with Protein A/G beads at room temperature.

### UbiCREST

Beas2B cells were transiently transfected with *NALP7*-V5/His mammalian expression vectors, and Ub-*NALP7*-V5 was V5 immunoprecipitated. Ub-NALP7-V5 beads were equally divided among 9 tubes prior to addition of DUBs per the manufacturer's protocol (Boston Biochem). Following incubation, the supernatant was removed; remaining bound substrate was eluted with the addition of 1x SDS protein sample buffer and analysed by immunoblotting.

### Preparation of ubiquitinated NALP7 substrate

HeLa cells were transiently transfected with *NALP7*-V5/His and HA-*ubiquitin* mammalian expression vectors (2.5 μg of each plasmid per 10 cm dish) for 24–48 h. Before collecting, cells were treated with MG132 (20 μM) and leupeptin (50 μM) for 1 h. Cells were lysed in a denaturing buffer (50 mM Tris, pH 7.5, 150 mM sodium chloride, 1% SDS and 0.25% Triton X100) supplemented with phosphatase and protease inhibitors (50 mM sodium fluoride, 2 mM sodium orthovanadate, 10 mM sodium pyrophosphate, 1 mM PMSF and 2 μM leupeptin), including DUB specific inhibitors (20 μM PR-619 and 5 mM *N*-ethylmaleimide). Lysates were immediately heated to 95 °C for 10 min, followed by sonication. Lysates were then diluted 1:4 in dilution buffer (50 mM Tris, pH 7.5, 150 mM sodium chloride, 0.25% Triton X100, 2 mM EDTA) supplemented with the above mentioned phosphatase, protease and DUB specific inhibitors. Lysates were clarified, and protein concentrations were determined. Lysates were further diluted with dilution buffer to decrease the final SDS concentration to 0.1% or less. Immunoprecipitation was performed by adding 1 μl of V5 antibody per 1 mg of lysate and rocking for 1 h at 4 °C. 12 μl of a 50% slurry of protein A/G resin was added per 1 mg of lysate and rocked for 1 h at 4 °C. Lysates and resin were washed twice with dilution buffer and then thrice with DUB reaction buffer (50 mM Tris, pH 7.5, 150 mM sodium chloride, 0.25% Triton X100, 25 mM potassium chloride, 5 mM magnesium chloride and 1 mM DTT). Finally, resin was resuspended as a 30% slurry in reaction buffer.

### *In vitro* DUB assay

With NALP7 substrate—purified, recombinant STAMBP (R&D Systems, E-548B), STAM (R&D Systems, E-550), or vehicle control were pre-incubated for 20 min at room temperature in a total volume of 5 μl of DUB reaction buffer, with inhibitors or vehicle control as indicated. Ubiquitinated NALP7 substrate (30% slurry of agarose-V5-protein complexes in DUB reaction buffer) was then added to the reaction mixture that was further incubated at 37 °C for up to 1 h. Reactions were stopped by the addition of 8 μl of 4 × SDS protein sample and immediate heating to 95 °C for 2 min. Samples were analysed by immunoblotting. Purified, recombinant K63-linked di-ubiquitin (Ub2) in 10 μl of DUB reaction buffer was added to bring the final volume to 20 μl, before incubation at 37 °C for 2 h. Reactions were stopped and analysed as previously described.

### Molecular docking studies and compound design

Using a library containing greater than 500,000 experimental compounds, molecular docking analysis and score-ranking operations were performed *in silico* to assess potential ligands that might fit the predicted STAMBP—Ub binding groove 3D structural model. These docking experiments were conducted using the LibDock program from Discovery Studio 3.5. On the basis of docking and best-fit analysis, we selected 4-hydroxyquinazoline as a backbone and further screened its analogs *in silico*. The top 10 score-ranking molecules were synthesized by a contract research organization (ChemDiv) and further tested in cell based and *in vitro* models.

### Off-target DUB screening

BC-1471 was tested for off-target DUB activity by a contract research organization (Ubiquigent) using their DUB*profiler* service. Briefly, 38 distinct DUBs are pre-incubated with vehicle control or BC-1471 before assay with ubiquitin-rhodamine substrate. Data were converted to per cent activity by dividing the fluorescence of the BC-1471-treated condition by the related control for each DUB.

### MMP activity assay

Recombinant MMP-1, MMP-2, MMP-8 and MMP-9 were obtained from Anaspec and activity was assayed using the SensoLyte Fluorimetric 520 Generic MMP Activity Kit (Anaspec, Fremont CA) according to the manufacturer's instructions. Briefly, MMP-2 and MMP-8 were incubated with 1 mM AMPA (4-aminophenylmercuric acetate) at 37 °C for 1 h to activate. In each well we added 30, 3, 30 and 60 ng of MMP-1, MMP-2, MMP-8 and MMP-9, respectively. Either DMSO, GM6001, or BC-1471 were added prior to the addition of the cleavable fluorescent MMP substrate. Relative fluorescence was measured at 1 h.

### Compound stability

Stability testing was performed by a contract research organization (Cyprotex) using mass spectrometry. Briefly, BC-1471 was added to cell culture media and incubated at 37 °C. At the indicated time points, an aliquot was removed, mixed with Stop Solution and analysed by LC/MS/MS to quantitate the remaining product. Data were converted to per cent remaining by dividing by the time zero concentration value.

### Human lung slices

Donor human lungs not accepted for transplant were obtained through the University of Pittsburgh Committee for Oversight of Research and Clinical Training Involving Decedents (CORID). Donor medical records were de-identified and IRB approval is not required to access these tissues. Organs were considered appropriate for study if there was no evidence of parenchymal lung disease, gas exchange was within normal limits before collecting, and organs could be processed with <6 h cold ischaemic time. Localized lesions (for example, solitary nodules) were avoided during tissue selection. Single lung segments were dissected and warmed in a weighted plastic bag in a 37 °C water bath for 30 min. Two per cent low melting point agarose in PBS (Invitrogen Ultrapure) is also maintained at 37 °C. The lung segments were filled with agarose by instillation into airways via syringe with 18 G canula and inspected for appropriate expansion, followed by airway clamping. Tissue is placed in a bag and submerged in ice for 30 min or until agarose has set. Tissue is cut to block size and sliced in ice cold saline with a vibratome (Leica VT 1200) at slice thickness of 300 μm. Uniform slices were sectioned into 1 cm × 1 cm sections and cultured in RPMI containing Pen/strep and amphotericin B without serum in 12-well dishes at 37 °C in a tissue incubator with 5% CO_2_. Media was changed after 2 h and experiments performed in 1 ml media after overnight incubation.

### FLICA assay

THP-1 cells (1 × 10^6^ cells per ml) were pre-treated with BC-1471 (10 μg ml^−1^) for 16 h prior to addition of LPS (500 ng ml^−1^), Pam3CSK4 (100 ng ml^−1^) or control for 6 h. FAM-FLICA reagent (Immunochemistry Technologies, 98) was added 4 h before the end of the assay, prepared according to the manufacturer's protocol. Cells were washed and resuspended in wash buffer, followed by flow cytometry analysis using the 488 nm excitation channel.

### Statistical analysis

All statistical analysis was performed using Graph Pad Prism 7 software for Mac OS X, version 7.0a.

### Data availability

The data that support the findings of this study are available from the corresponding author upon reasonable request.

## Additional information

**How to cite this article:** Bednash, J. S. *et al*. Targeting the deubiquitinase STAMBP inhibits NALP7 inflammasome activity. *Nat. Commun.* **8,** 15203 doi: 10.1038/ncomms15203 (2017).

**Publisher's note**: Springer Nature remains neutral with regard to jurisdictional claims in published maps and institutional affiliations.

## Supplementary Material

Supplementary Information Supplementary Figures and Supplementary Tables
