# Supplementary material for: Targeting the deubiquitinase STAMBP inhibits NALP7 inflammasome activity
Source: Nat Commun. 2017 May 11;8:15203. doi: 10.1038/ncomms15203 (PMC5437278; doi:10.1038/ncomms15203)
Supplement: Supplementary Information — Supplementary Figures and Supplementary Tables [file ncomms15203-s1.pdf]

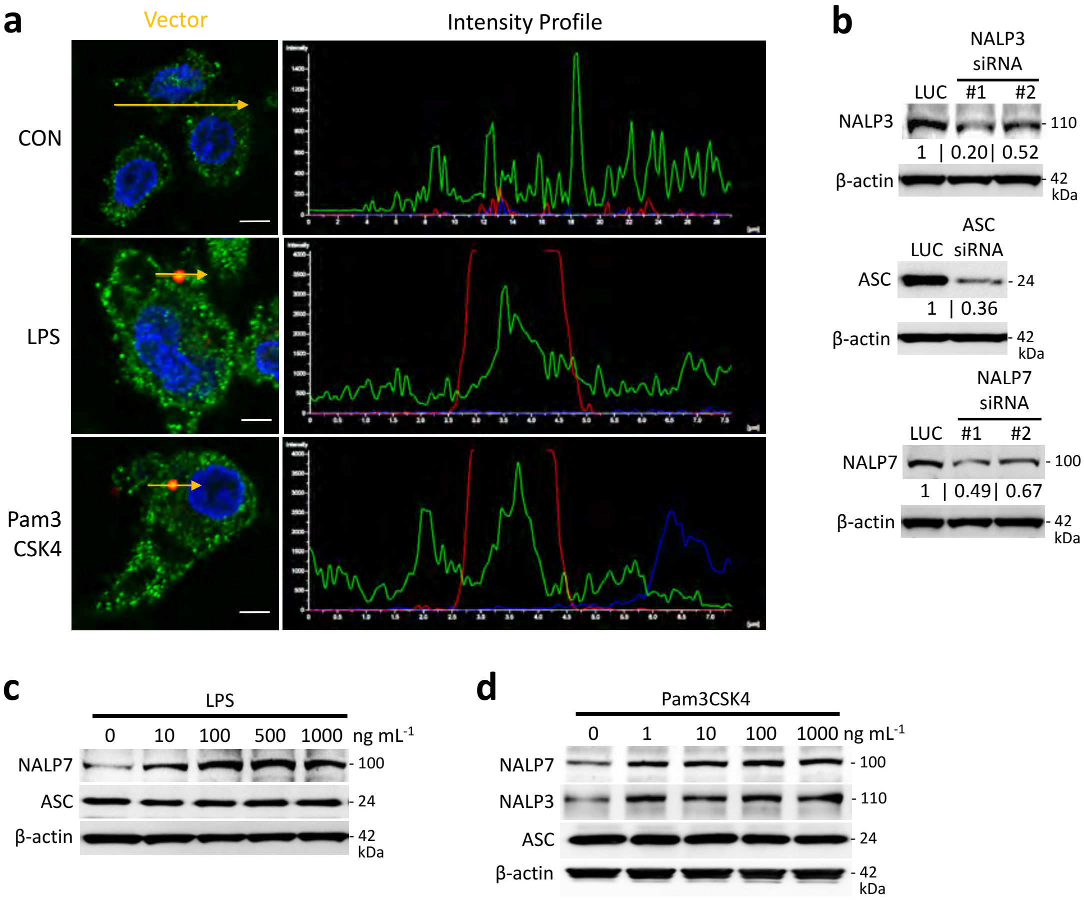

Supplementary Figure 1. Cellular expression of inflammasome components after stimuli. (a) THP-1 macrophages that formed an ASC-speck in Fig. 1b were further analyzed by immunofluorescence intensity profiling. Intensity was graphed as relative fluorescent units (RFUs) across the vector depicted by the yellow arrow. Cells exposed to LPS or Pam3CSK4 displayed formation of an ASC-speck (red) with co-localization of increased intensity NALP7 signal (green). Original magnification was 100x, scale bars = 10 µm. (b) Immunoblot analysis confirmed knockdown of NALP3, ASC, or NALP7 by siRNA or a control luciferase (LUC) in THP-1 cells assayed in Fig. 1c. Relative abundance compared to control noted below as determined by densitometry using ImageJ software. (c, d) Immunoblot analysis of NALP7, NALP3, and ASC in lysates of THP-1 cells after (c) LPS or (d) Pam3CSK4 exposure for 16 h.

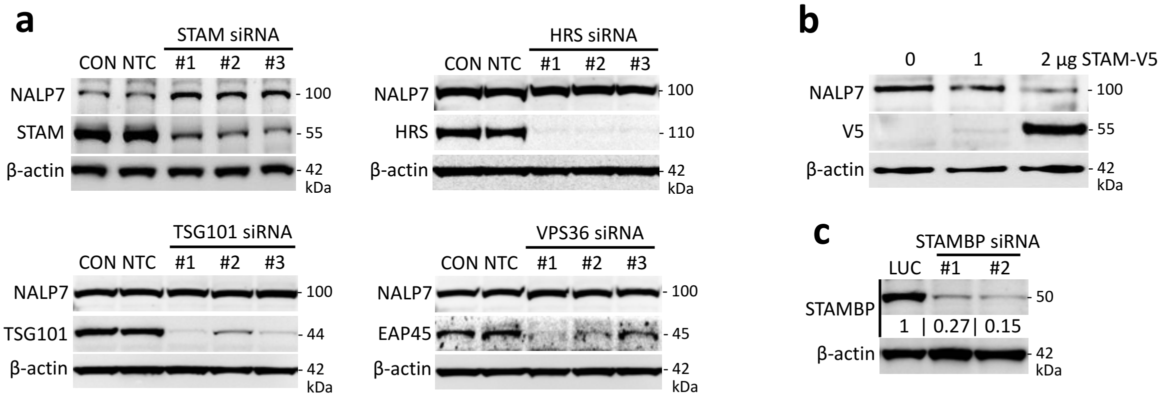

Supplementary Figure 2. STAM modulates NALP7 abundance. (a) *STAM* knockdown with siRNA increases NALP7 protein abundance in Beas2B cells compared to untreated control (CON), or a nontargeting control (NTC) siRNA. *HRS, Tsg101,* and *VPS36* knockdown with siRNA did not impact NALP7 protein abundance. Note: EAP45 is the protein product of the VPS36 gene. (b) Overexpression of *STAM*-V5 plasmid in Beas2B cells decreased immunoreactive NALP7. (c) Immunoblot analysis confirmed knockdown of STAMBP by siRNA or a control luciferase (LUC) in THP-1 cells assayed in Fig. 4i. Relative abundance compared to control noted below as determined by densitometry using ImageJ software.

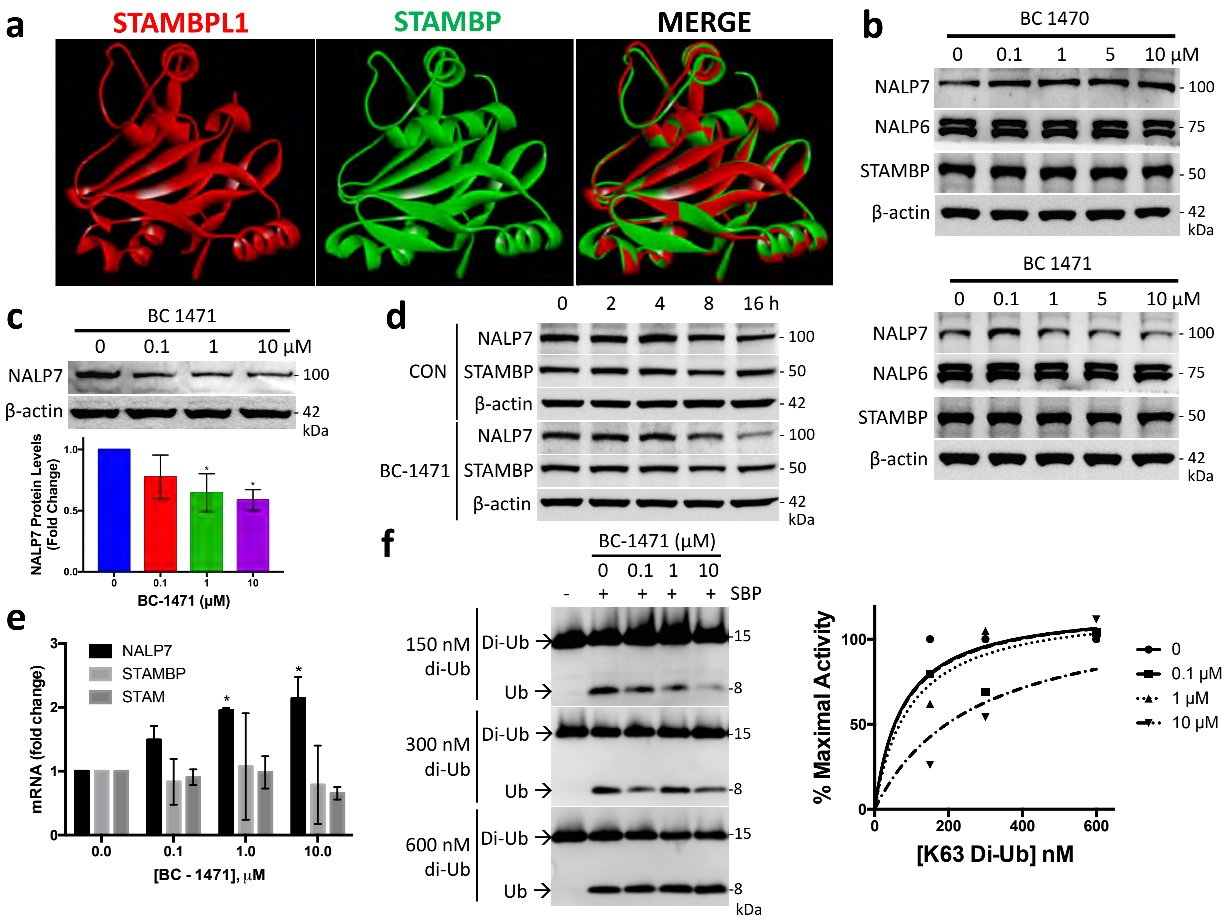

Supplementary Figure 3. Small molecule antagonism of NALP7. (a) STAMBPL1 (2ZNV.pdb) and STAMBP (3RZU.pdb) in a structural homology model (merge). (b) Phenotypic screening of NALP7 small molecule antagonists. (c) Endogenous NALP7 protein abundance is decreased in THP-1 cells exposed to BC-1471. *Below,* densitometric analysis of relative NALP7 protein abundance as determined by ImageJ software. Data shown as mean ± SD (n=3). **P* < 0.05 compared to control. (d) Endogenous NALP7 half-life is decreased with BC-1471 pre-treatment (10μM for 2 h) compared to control in Beas2B cells. (e) NALP7, STAMBP and STAM mRNA with BC-1471 treatment for 16 h as analyzed by qPCR in THP-1 cells. Data shown as mean fold change ± SD by ΔΔCq analysis (n=3). (f) *In vitro* DUB assay as performed in Fig. 5d. Right: Relative mono-Ub was quantified by densitometry and fit to multiple inhibition models using GraphPad Prism software (n=2). (c) One-way and (e) two-way analysis of variance (ANOVA) with post-hoc Dunnett's multiple comparisons test.

a

	BC-1471 10 μM		BC-1471 10 μM		BC-1471 10 μM
USP1/UAF1	116	USP19	94	OTU1	110
USP2	104	USP20	95	OTUB2	100
USP4	115	USP21	101	OTUD3	98
USP5	101	USP25	120	OTUD5	111
USP5 (+Ub@Kd)	98	USP28	102	OTUD6A	97
USP5 (+Ub@Bmax)	104	USP30	92	OTUD6B	93
USP6	101	USP35	107	Cezanne	89
USP7	143	USP36	100	AMSH-LP	89
USP8	98	USP45	108	AMSH-LP (+Zinc)	113
USP9x	87	CYLD	97	Ataxin3	95
USP11	108	UCHL1	89	Ataxin3L	105
USP14	113	UCHL3	96	JOSD1	110
USP15	100	UCHL5	103	JOSD2	103
USP16	98	BAP1	96		

b

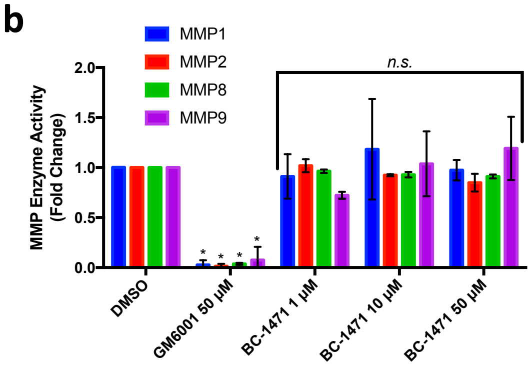

c

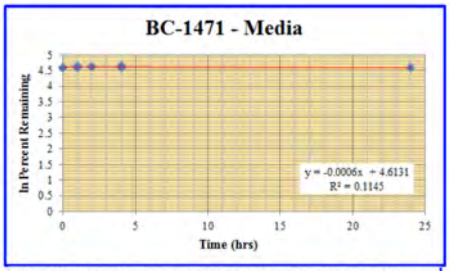

Time (Hrs)	% Remaining Replica 1	% Remaining Replica 2	Average
24	98.7%	99.4%	99.1%
4	99.7%	103.6%	101.7%
2	100.6%	101.4%	101.0%
1	97.9%	103.0%	100.5%
0	100%	100%	100%

d

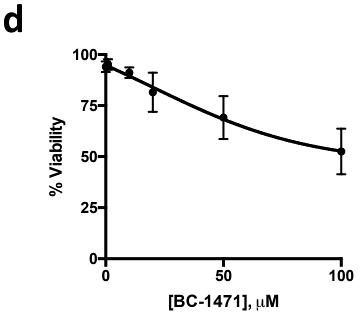

Supplementary Figure 4. Characterization of BC-1471. (a) BC-1471 treatment did not significantly inhibit the enzymatic activity of any of the tested DUBs in a high-throughput *in vitro* screen for off-target DUB inhibition. Data shown as mean percent of untreated control DUB activity (*n*=2). (b) BC-1471 treatment did not significantly inhibit the enzymatic activity of any of the tested MMPs in a fluorescent MMP activity assay. Data shown as MMP activity measured by relative fluorescence compared to the DMSO control (*n*=3). (c) BC-1471 was stable in RPMI media, as measured by mass spectrometry (*n*=2). (d) Percent viability of THP-1 cells treated with BC-1471 for 16 h as determined by trypan blue staining. Data represents mean ± SD with standard curve fit by nonlinear regression (*n*=3). (b) Two-way analysis of variance (ANOVA) with post-hoc Dunnett's multiple comparisons test.

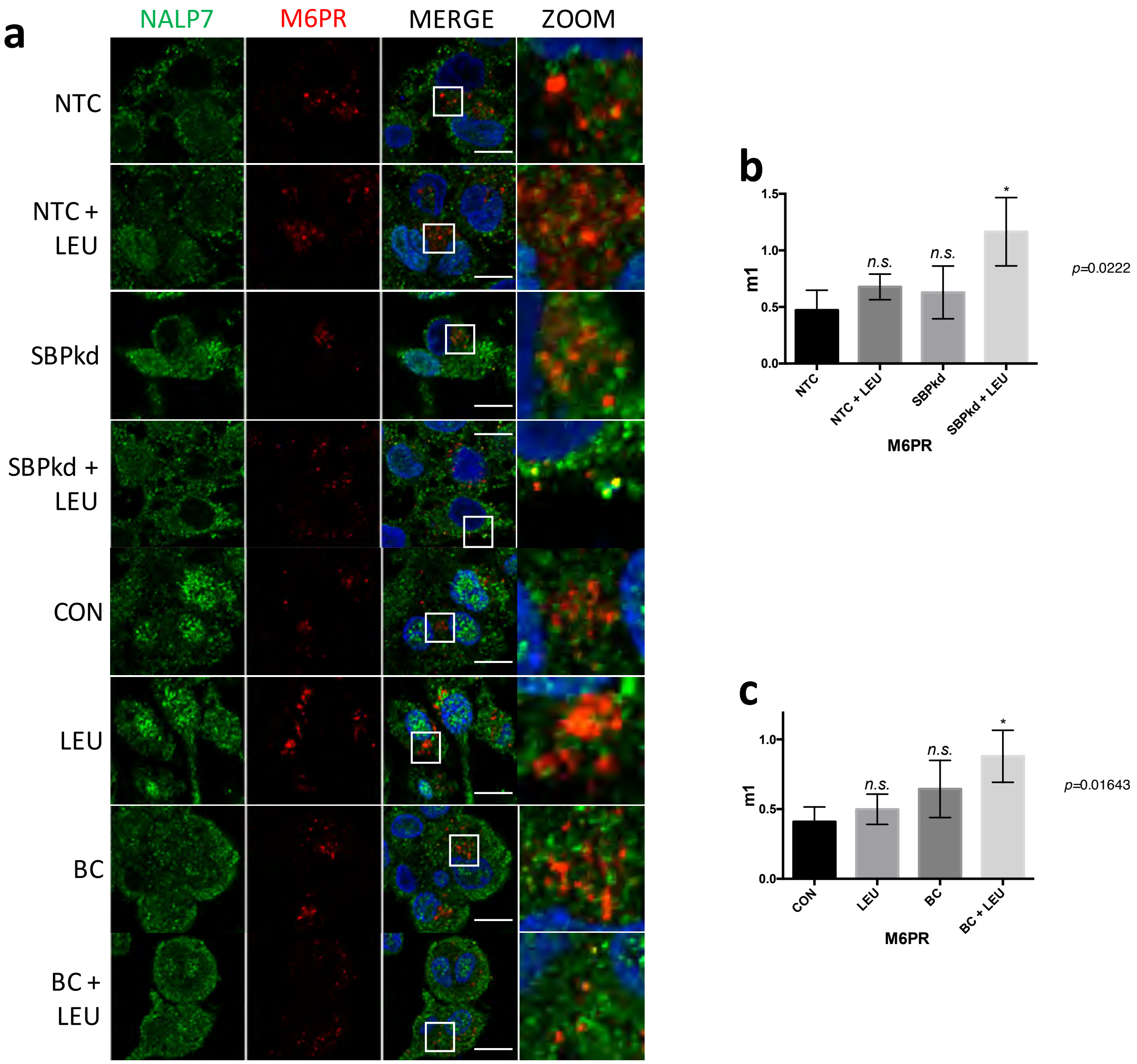

Supplementary Figure 5. BC-1471 relocates NALP7 . (a) Representative images obtained by immunofluorescence co-staining of NALP7 (green), M6PR (red) and DAPI (blue) in PMA-differentiated THP-1 cells following STAMBP knockdown (SBP kd) with siRNA, nontargeting control plasmid (NTC), BC-1471 (BC) treatment (10 µM for 16 h), leupeptin (LEU) treatment (50 µM for 2 h), or a combination thereof. Original magnification was 100x, scale bars = 10 µm. (b,c) Colocalization analysis of images obtained as in Fig S5a. Data represents mean ± SD for the m1 colocalization coefficient of the red-green channel pair (*n*=3). (b, c) One-way analysis of variance (ANOVA) with post-hoc Dunnett's multiple comparisons test.

Supplementary Figure 6. Uncropped Western blots.

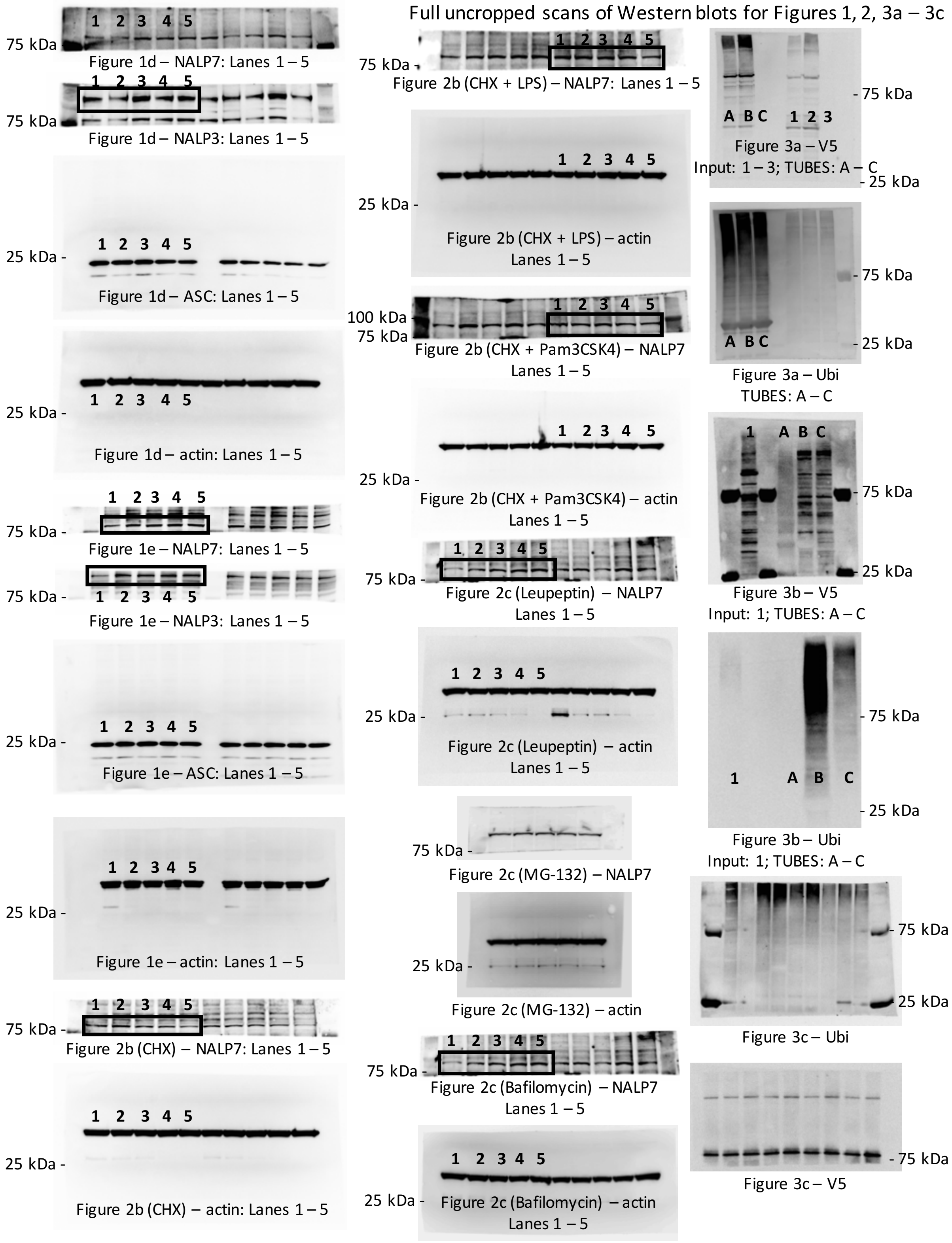

Supplementary Figure 6 (cont). Uncropped Western blots.

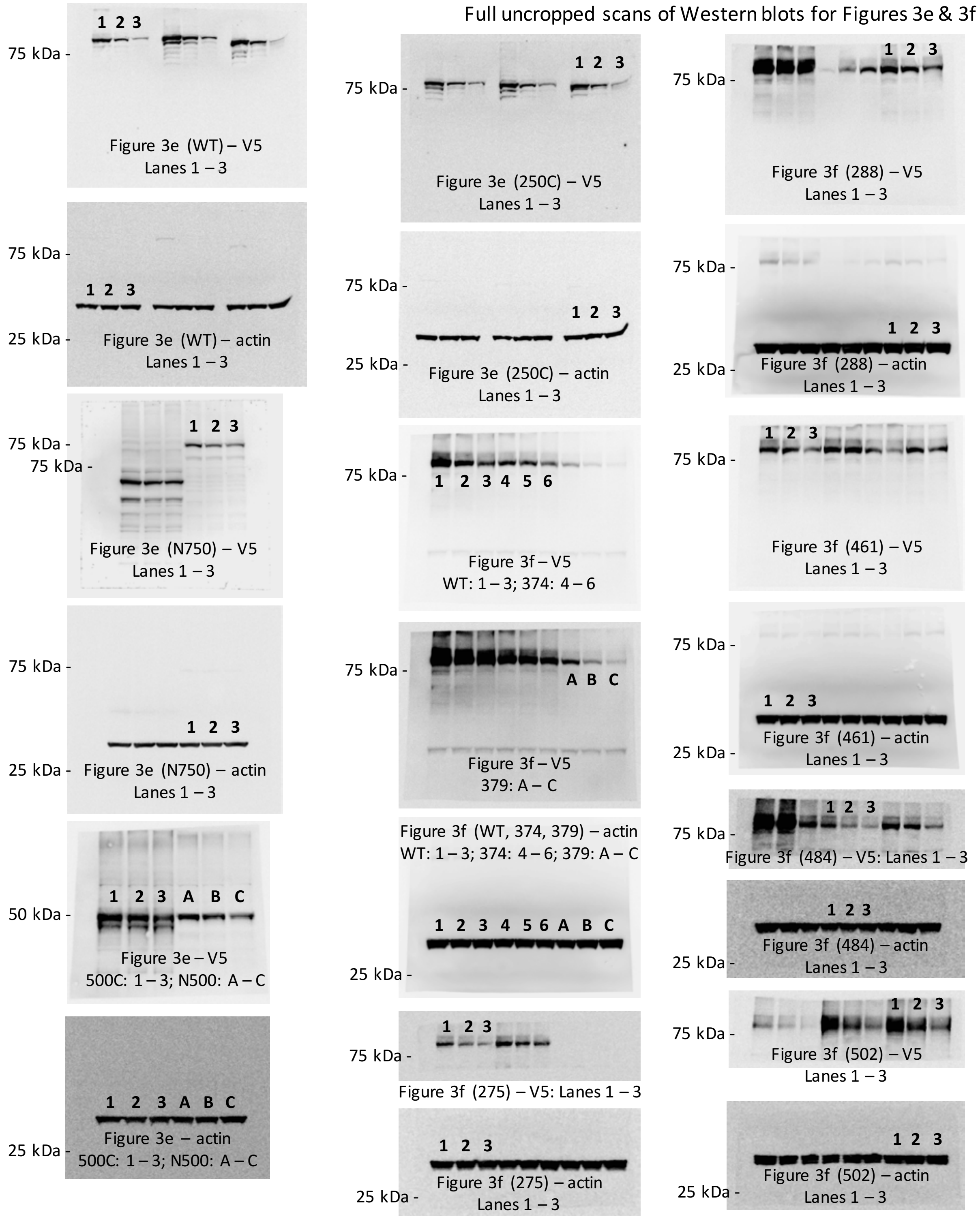

Supplementary Figure 6 (cont). Uncropped Western blots.

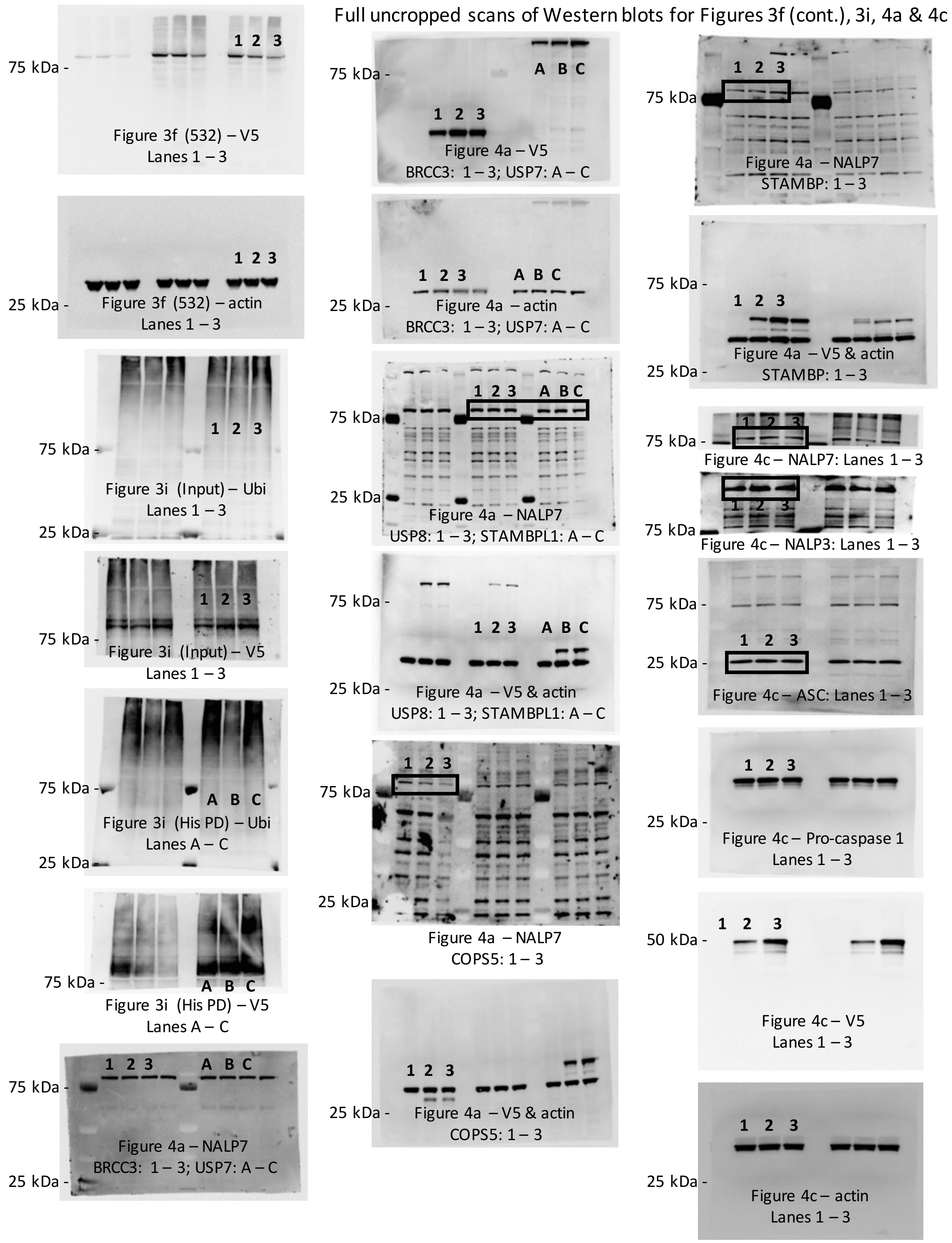

Supplementary Figure 6 (cont). Uncropped Western blots.

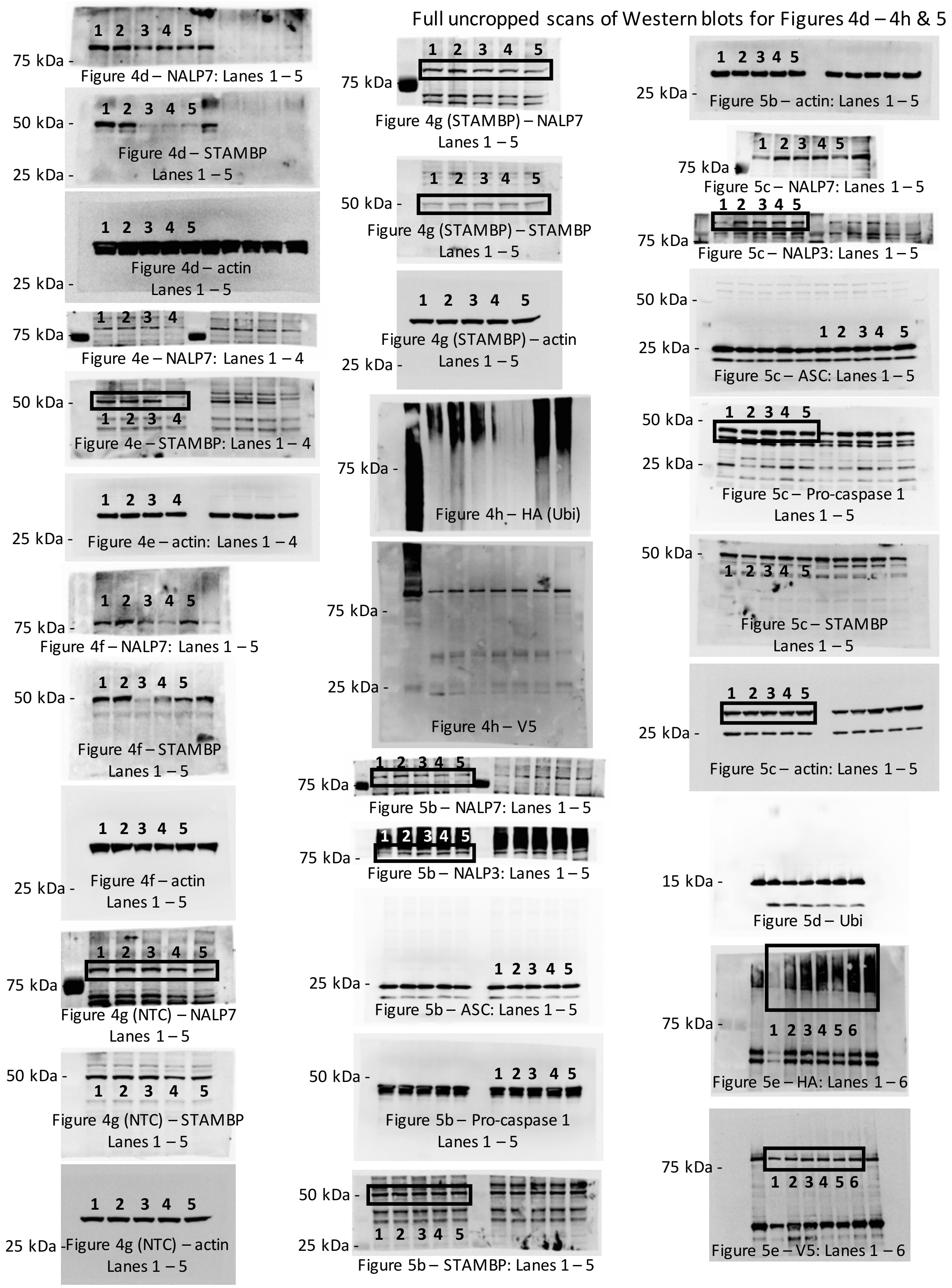

Supplementary Figure 6 (cont). Uncropped Western blots.

Full uncropped scans of Western blots for Figure S1 & S2

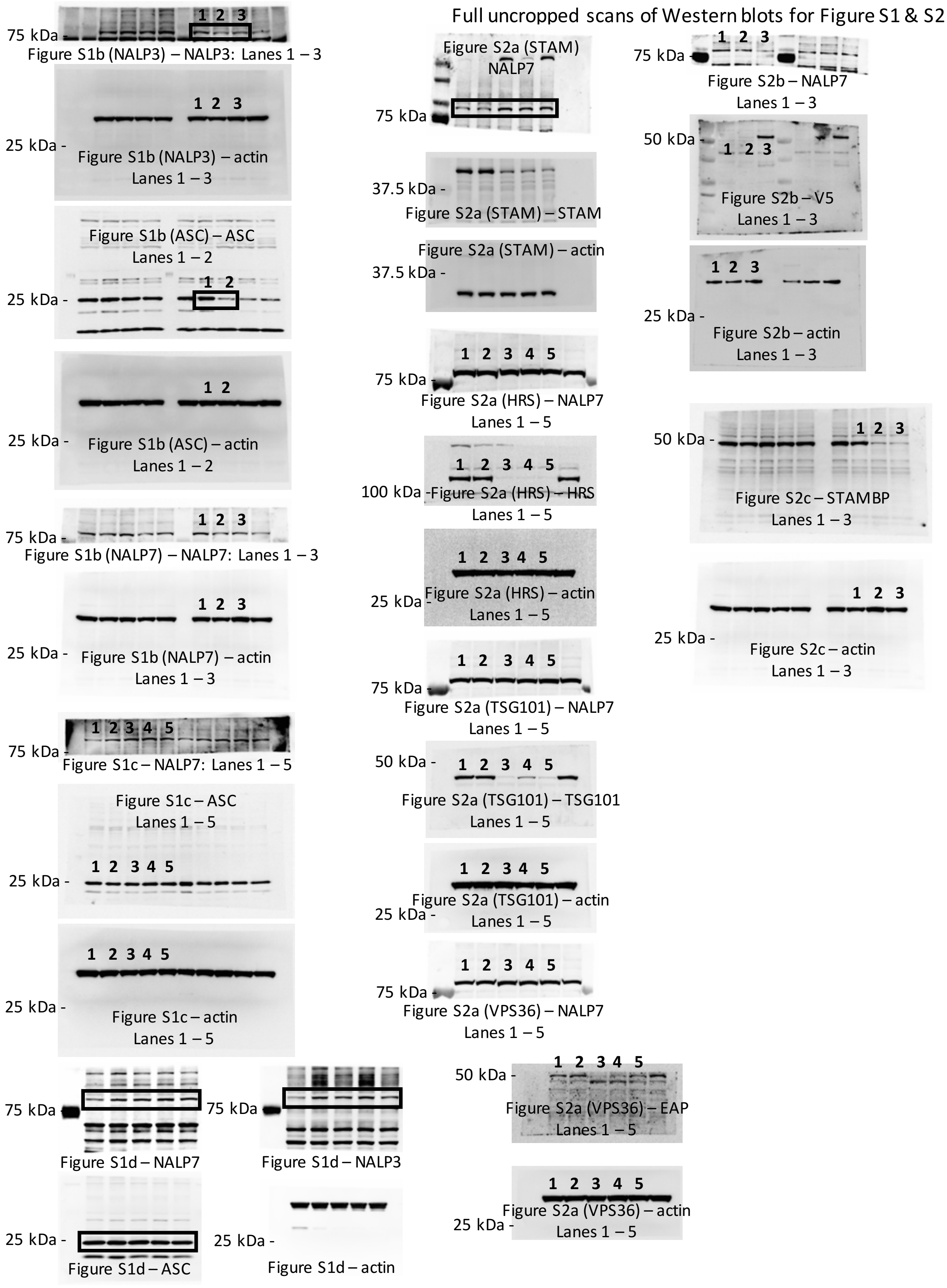

Supplementary Figure 6 (cont). Uncropped Western blots.

Full uncropped scans of Western blots for Figure S3

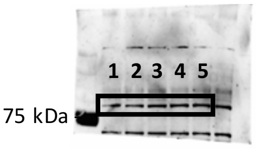

Figure S3b (1470) – NALP7
Lanes 1 – 5

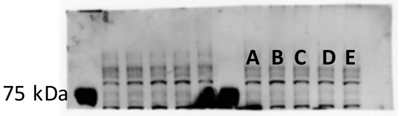

Figure S3b (1471) – NALP7
Lanes A – E

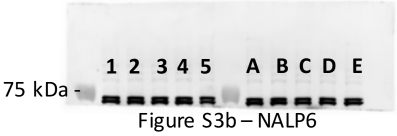

Figure S3b – NALP6
BC1470: 1 – 5; BC1471: A – E

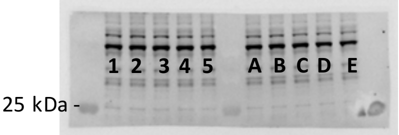

Figure S3b – STAMBP
BC1470: 1 – 5; BC1471: A – E

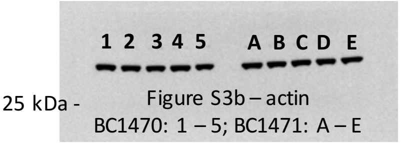

Figure S3b – actin
BC1470: 1 – 5; BC1471: A – E

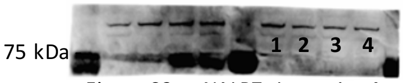

Figure S3c – NALP7: Lanes 1 – 4

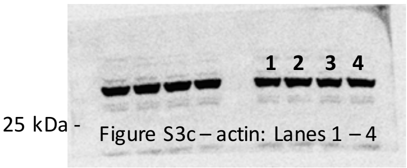

Figure S3c – actin: Lanes 1 – 4

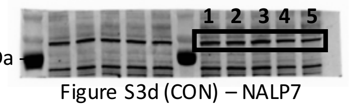

Figure S3d (CON) – NALP7
Lanes 1 – 5

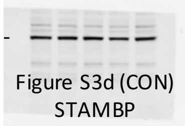

Figure S3d (CON)
STAMBP

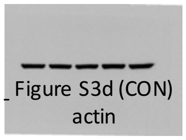

Figure S3d (CON)
actin

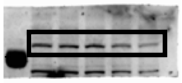

Figure S3d (BC-1471) – NALP7

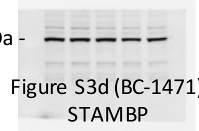

Figure S3d (BC-1471)
STAMBP

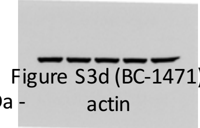

Figure S3d (BC-1471)
actin

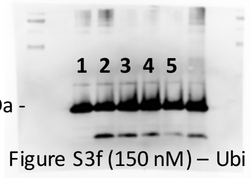

Figure S3f (150 nM) – Ubi
Lanes 1 – 5

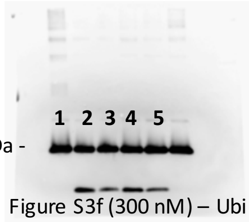

Figure S3f (300 nM) – Ubi
Lanes 1 – 5

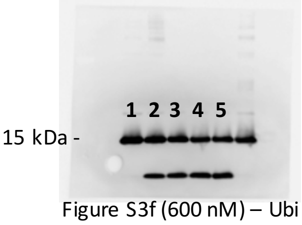

Figure S3f (600 nM) – Ubi
Lanes 1 – 5

Supplementary Table 1. List of primer sequences.

**PLASMID CLONING**		
Target	Forward	Reverse
NALP7 for topo	CAC CAT GTC TGA CCA TGG AGA TGT GAG	GCA AAA AAA GTC ACA GCA CGG AGG
STAMBP for topo	CAC CAT GTC TGA CCA TGG AGA TGT GAG	TCG AAG GTC TGT GAT GGT CAC TGC
**qPCR PRIMERS**		
Target	Forward primer	Reverse primer
NALP7	CTG CAG ATG TTG TCG TTG G	CTT GCT GAC AAC CAA GAC AG
Pro-IL-1beta	CTC GCC AGT GAA ATG ATG G	TGG AAG GAG CAC TTC ATC TG
STAMBP	CCT CTA TAA CAA GTA TAT CAC GCT C	CTT TGG GAA ATG CAA TCT CCT
STAM	ACT TTG AAG CTG CTG AAG AC	TCC ACC AGT TAG GAT CAC TG
GAPDH	GCA TCC TGG GCT ACA CTG AG	AAG TGG TCG TTG AGG GCA AT
**TRUNCATION MUTANT PRIMERS**		
WT NALP7 forward	CAC CAT GTC TGA CCA TGG AGA TGT GAG	
WT NALP7 reverse	GCA AAA AAA GTC ACA GCA CGG AGG	
N500 (reverse)	TAC GTC CCC GAT GTC CCA GG	
N750 (reverse)	CAG CAT CAT CGT GCG TTC CCA	
250C (forward)	CAC CAT GGT GGT CGA TGG CC	
500C (forward)	CAC CAT GCA GAA GCT GCT TTC CG	
**SITE-DIRECTED MUTAGENESIS PRIMERS**		
Target	Forward	Reverse
KKK275RRR	GCG GGG ACT GGG AGA GAA GAA GAC CGG TGC CCG TCC TC	GAG GAC GGG CAC CGG TCT TCT TCT CTC CCA GTC CCC GC
KRK288RRR	GGG GAG TTT GCT GAG AAG ACG GAT GTT ACC CAG GG	CCC TGG GTA ACA TCC GTC TTC TCA GCA AAC TCC CC
K374R	GTG CAC GAC TCT GAG ACT GCA GAT GGA GAA G	CTT CTC CAT CTG CAG TCT CAG AGT CGT GCA C
K379R	GCA GAT GGA GAG AGG GGA GGA CCC G	CGG GTC CTC CCC TCT CTC CAT CTG C
K461R	GAC AGA GTC TCC AGA GGC TGC TAC TC	GAG TAG CAG CCT CTG GAG ACT CTG TC
K484R	CTA CGC CCT GGA GAG AGA GGA GGG GGA GG	CCT CCC CCT CCT CTC TCT CCA GGG CGT AG
K502R	GGG ACG TAC AGA GAC TGC TTT CCG G	CCG GAA AGC AGT CTC TGT ACG TCC C
K532R	GAG AAG AGA GCC AGA GAG TTG GAG GCC AC	GTG GCC TCC AAC TCT CTG GCT CTC TTC TC

Supplementary Table 2. List of DsiRNA sequences.

Target		Sequence (Sense)	Sequence (Antisense)
NLRP3	1	rGrGrA rArGrA rCrArC rCrArG rGrArC rArArU rGrArC rArGC A	rUrGrC rUrGrU rCrArU rUrGrU rCrCrU rGrGrU rGrUrC rUrUrC rCrUrC
	2	rGrGrU rGrUrU rGrGrA rArUrU rArGrA rCrArA rCrUrG rCrAA C	rGrUrU rGrCrA rGrUrU rGrUrC rUrArA rUrUrC rCrArA rCrArC rCrUrG
ASC	1	rGrCrG rArGrG rGrUrC rArCrA rArArC rGrUrU rGrArG rUrGG C	rGrCrC rArCrU rCrArA rCrGrU rUrUrG rUrGrA rCrCrC rUrCrG rCrGrA
NLRP7	1	rGrGrU rGrCrA rArGrA rArArU rArGrA rUrArA rUrCrC rUrGA G	rCrUrC rArGrG rArUrU rArUrC rUrArU rUrUrC rUrUrG rCrArC rCrUrG
	2	rGrGrA rUrArA rUrArA rArGrU rUrArG rArGrC rArCrU rCrUC G	rCrGrA rGrArG rUrGrC rUrCrU rArArC rUrUrU rArUrU rArUrC rCrCrU
STAMB P	1	rGrUrA rGrCrU rCrArG rArArA rUrUrA rArGrA rArArG rArAT G	rCrArU rUrCrU rUrUrC rUrUrA rArUrU rUrCrU rGrArG rCrUrA rCrUrU
	2	rGrGrA rGrUrU rGrArG rArUrU rArUrC rCrGrA rArUrG rGrCA T	rArUrG rCrCrA rUrUrC rGrGrA rUrArA rUrCrU rCrArA rCrUrC rCrArG
	3	rGrCrU rCrUrU rArCrC rArGrA rUrGrA rUrGrU rUrGrC rCrAG A	rUrCrU rGrGrC rArArC rArUrC rArUrC rUrGrG rUrArA rGrArG rCrArG
STAM	1	rArGrA rArUrU rGrArU rArCrA rArGrG rCrUrA rUrUrU rGrUC T	rArGrA rCrArA rArUrA rGrCrC rUrUrG rUrArU rCrArA rUrUrC rUrUrU
	2	rArGrA rGrUrG rArArC rCrArC rArArA rGrArU rCrCrU rCrAC G	rCrGrU rGrArG rGrArU rCrUrU rUrGrU rGrGrU rUrCrA rCrUrC rUrUrC
	3	rArGrA rArUrG rArUrC rCrArC rArGrC rUrUrA rGrUrC rUrAA T	rArUrU rArGrA rCrUrA rArGrC rUrGrU rGrGrA rUrCrA rUrUrC rUrUrA
HRS	1	rGrCrC rUrUrA rCrArA rCrArU rGrCrA rGrArA rUrCrU rCrAT G	rCrArU rGrArG rArUrU rCrUrG rCrArU rGrUrU rGrUrA rArGrG rCrUrG
	2	rArGrA rArUrU rCrArA rArGrA rGrArG rCrGrA rUrGrC rCrAT G	rCrArU rGrGrC rArUrC rGrCrU rCrUrC rUrUrU rGrArA rUrUrC rUrGrG
	3	rGrGrA rGrUrA rArCrA rCrUrA rCrArU rArCrA rGrUrU rCrAC C	rGrGrU rGrArA rCrUrG rUrArU rGrUrA rGrUrG rUrUrA rCrUrC rCrGrG
TSG101	1	rGrCrC rUrUrG rArArA rCrGrA rArCrA rGrArA rGrArA rGrAC C	rGrGrU rCrUrU rCrUrU rCrUrG rUrUrC rGrUrU rUrCrA rArGrG rCrArU
	2	rUrGrG rUrGrU rCrCrA rArGrU rArCrA rArArU rArCrA rGrAG A	rUrCrU rCrUrG rUrArU rUrUrG rUrArC rUrUrG rGrArC rArCrC rArUrU
	3	rArGrA rUrCrA rArGrA rArGrU rArGrC rCrGrA rGrGrU rUrGA T	rArUrC rArArC rCrUrC rGrGrC rUrArC rUrUrC rUrUrG rArUrC rUrArA
Vps36	1	rGrGrA rArUrA rGrCrU rArArC rCrCrA rGrUrU rArCrC rArGA G	rCrUrC rUrGrG rUrArA rCrUrG rGrGrU rUrArG rCrUrA rUrUrC rCrCrA
	2	rCrCrU rArGrC rCrArA rArGrA rArArG rGrUrU rGrCrU rGrCT T	rArArG rCrArG rCrArA rCrCrU rUrUrC rUrUrU rGrGrC rUrArG rGrArG
	3	rGrGrC rUrArA rUrArC rUrGrC rArUrG rUrArG rArUrU rGrCT T	rArArG rCrArA rUrCrU rArCrA rUrGrC rArGrU rArUrU rArGrC rCrArG
